# Synthesis and In Vitro Biological Evaluation of Amphiphilic Derivatives of Carvacrol as Potent Antibacterial Agents

**DOI:** 10.3390/antibiotics15090927

**Published:** 2026-09-18

**Authors:** Danila A. Zadvornykh, Meiling Wang, Alevtina V. Bardasheva, Anna S. Pavlova, Olga D. Zakharova, Elena V. Dmitrienko, Lyudmila S. Koroleva, Wei Xie, Vladimir N. Silnikov

**Affiliations:** 1 Knorre Institute of Chemical Biology and Fundamental Medicine, Siberian Branch of Russian Academy of Sciences, Lavrentiev Avenue 8, Novosibirsk 630090, Russia; danila.zadvornykh@gmail.com (D.A.Z.); bardasheva_a@1bio.ru (A.V.B.); pavlova@1bio.ru (A.S.P.); isar@1bio.ru (O.D.Z.); elenad@1bio.ru (E.V.D.); koroleva@1bio.ru (L.S.K.); 2 School of Life and Health Sciences, Fuyao University of Science and Technology, Zhihui Avenue 104, Fuzhou 350109, China; wml@fyust.edu.cn (M.W.); william.xie@fyust.edu.cn (W.X.)

**Keywords:** cationic amphiphiles, carvacrol derivatives, DABCO hybrids, antibacterial agents, antibiotic resistance, RNA hydrolysis, hemolytic activity, cytotoxicity

## Abstract

Background/Objectives: The rapid emergence and spread of antibiotic-resistant bacterial strains represent a major global health threat, requiring the development of novel broad-spectrum antimicrobial agents. This study aims to design, synthesize, and evaluate a novel series of multitarget amphiphilic carvacrol derivatives to combat molecular resistance mechanisms by combining a natural monoterpene with polycationic ribonuclease scaffolds. Methods: A series of 16 dimeric carvacrol–1,4-diazabicyclo[2.2.2]octane (DABCO) hybrids, including both novel and previously reported derivatives, with varying central and lateral linkers were synthesized. In vitro antibacterial activity was evaluated against Gram-positive (*S. aureus*) and Gram-negative (*E. coli*, *S. enterica*, *C. freundii*, *P. aeruginosa*, *A. baumannii*) strains. Antibacterial action was examined through a time-kill kinetic assay against *S. aureus* at minimum inhibitory concentrations (MICs). RNA-hydrolyzing efficiency (using a FAM-labeled 17-mer ribooligonucleotide), hemolytic activity on rabbit red blood cells, and cytotoxicity (MTT assay) against RPMI8226, LMTK, and MEF cell lines were assessed. Results: Several compounds exhibited potent antibacterial activity, with MIC values ranging from 4 to 63 µM against most strains. The most active hybrid, DL4CAR-6, demonstrated broad-spectrum efficacy (MIC 4–16 µM against most strains) and outperformed the reference amphiphile DL4-12 against *P. aeruginosa* eightfold. Bacterial growth inhibition kinetic assay revealed that the cationic amphiphiles eradicate *S. aureus* faster than the reference antibiotic, indicating rapid membrane disruption. Most hybrids demonstrated pronounced RNA-hydrolyzing activity (up to 60% cleavage). The hemolytic assay showed less than 10% hemolysis for most compounds even at 500 µM. Furthermore, DL4CAR-6 displayed low cytotoxicity (IC_50_ 114–250 µM), with safety thresholds exceeding effective antibacterial doses. Conclusions: The newly developed carvacrol–DABCO amphiphilic hybrids represent a promising class of multitarget antibacterial agents. Their broad-spectrum efficacy, bactericidal kinetics, RNA-cleaving activity, low hemolytic toxicity, and favorable selectivity indices support their potential for further in vivo evaluation.

## 1. Introduction

The emergence, development, and spread of bacterial antibiotic resistance represent one of the most serious challenges humanity has faced over the past century. This phenomenon results in hundreds of thousands of deaths annually and is recognized by the World Health Organization as a major global health threat [1,2]. The rise of antibiotic-resistant bacterial strains is largely driven by the misuse of antibiotics without professional guidance and self-medication, particularly in low- and middle-income countries [3]. Additionally, the use of antimicrobial agents in livestock farming plays a critical role: 73% of all antibiotics sold worldwide are used in animals raised for food production [4,5,6].

One promising approach to combat antibiotic resistance is the use of multitarget antibacterial agents [7,8,9,10]. These compounds can simultaneously interact with fundamentally different cellular targets in bacteria, thereby significantly complicating the development of molecular resistance mechanisms.

**Figure 1:**
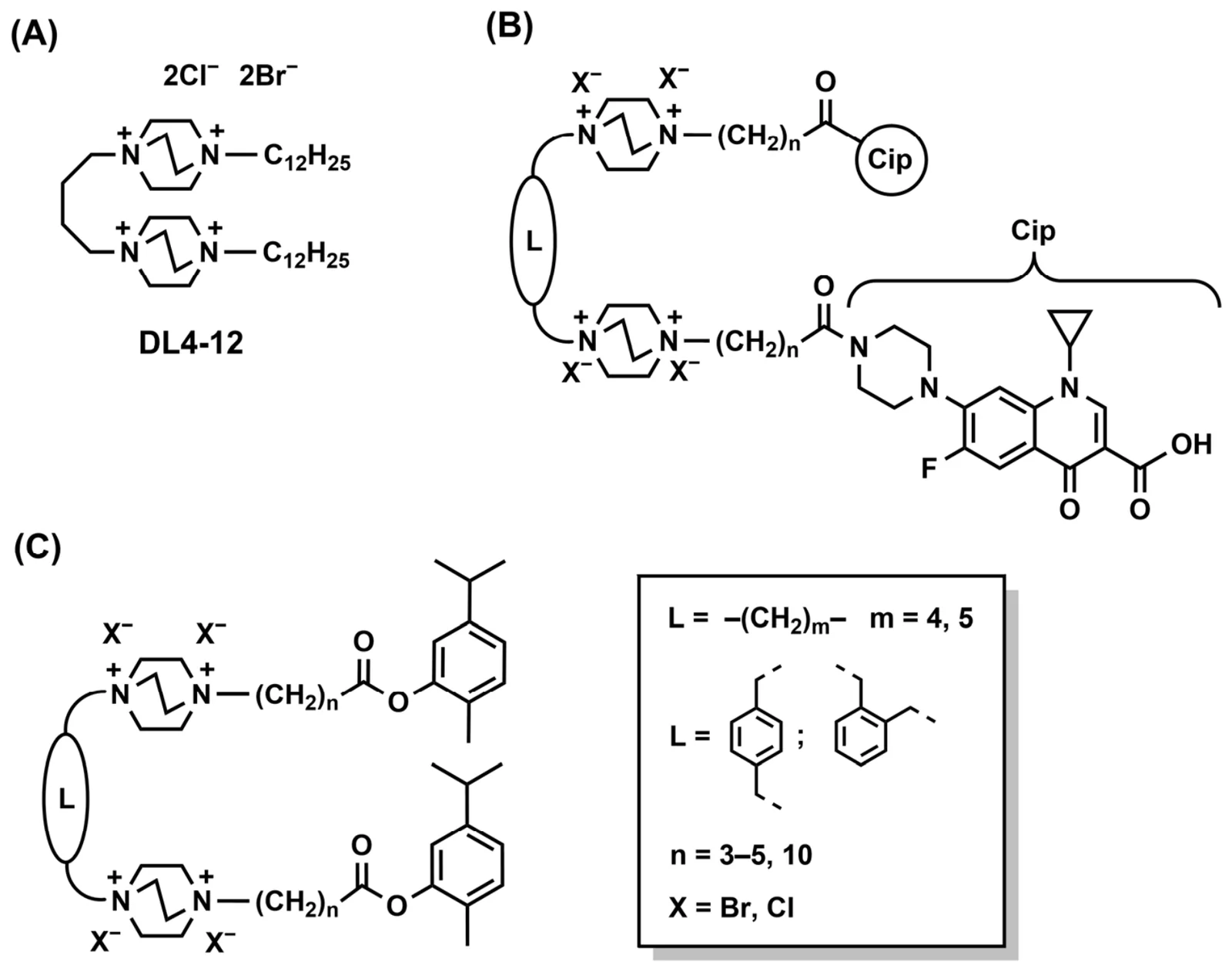
(**A**) Chemical structure of DABCO-based tetracationic amphiphile DL4-12; (**B**) Chemical structure of ciprofloxacin–DABCO hybrids; (**C**) Chemical structure of carvacrol–DABCO hybrids, discussed in this work. In the following, the linker group L connecting two DABCO residues will be referred to as the central linker, and the linker group connecting DABCO to the carvacrol moiety will be referred to as the lateral linker.

One approach to creating multitarget drugs is to combine known antibiotics with various amphiphiles [11,12,13,14,15]. Previously, we demonstrated that artificial ribonucleases based on 1,4-diazabicyclo[2.2.2]octane (DABCO)-derived cationic amphiphiles (Figure 1A) exhibit a broad spectrum of antibacterial activity [16,17,18]. In order to expand the range of bacterial targets, the antibiotic ciprofloxacin was incorporated into the structure of the cationic amphiphile (Figure 1B), yielding a hybrid compound [19]. A comparative study of the antibacterial mechanism of action of the parent antibiotic, the cationic amphiphile, and the hybrid structure was carried out using electron microscopy. It was found that the effect of the hybrid compound on *S. aureus* combined features of both ciprofloxacin and the cationic amphiphile components, but the effect was not simply additive. The characteristic membrane disruption typical of the cationic amphiphile DL4-12 (Figure 1A) was observed, while the ultrastructural changes in the cytoplasm were predominantly consistent with the effects of ciprofloxacin [20]. At the same time, the destruction of ribosomes associated with the ribonuclease activity of the original amphiphile [21] was not observed.

The presence of hydrophobic residues has been shown to be critical for RNA-hydrolyzing activity in prior studies [22].

In the present study, we aimed to develop multitarget antibacterial agents by combining previously studied polycationic amphiphiles, which exhibit RNA-hydrolyzing activity, with a hydrophobic natural monoterpene—carvacrol. The antibacterial activity of carvacrol is attributed to the destabilization of the bacterial membrane, reduction in membrane potential, dissipation of pH gradients, and disruption of lipid components in the bacterial cytoplasmic membranes [23,24].

The significance of this study lies in the structural integration of natural monoterpenes with polycationic scaffolds that possess intrinsic RNA-hydrolyzing and membranolytic activity. By simultaneously targeting the bacterial cell envelope and intracellular nucleic acids, these newly developed agents offer a promising, low-toxicity platform for the development of broad-spectrum antibacterials, a dual-action strategy that may potentially reduce the likelihood of rapid resistance emergence.

## 2. Results and Discussion

### 2.1. Design and Synthesis of Carvacrol–DABCO Hybrids

**Figure 2:**
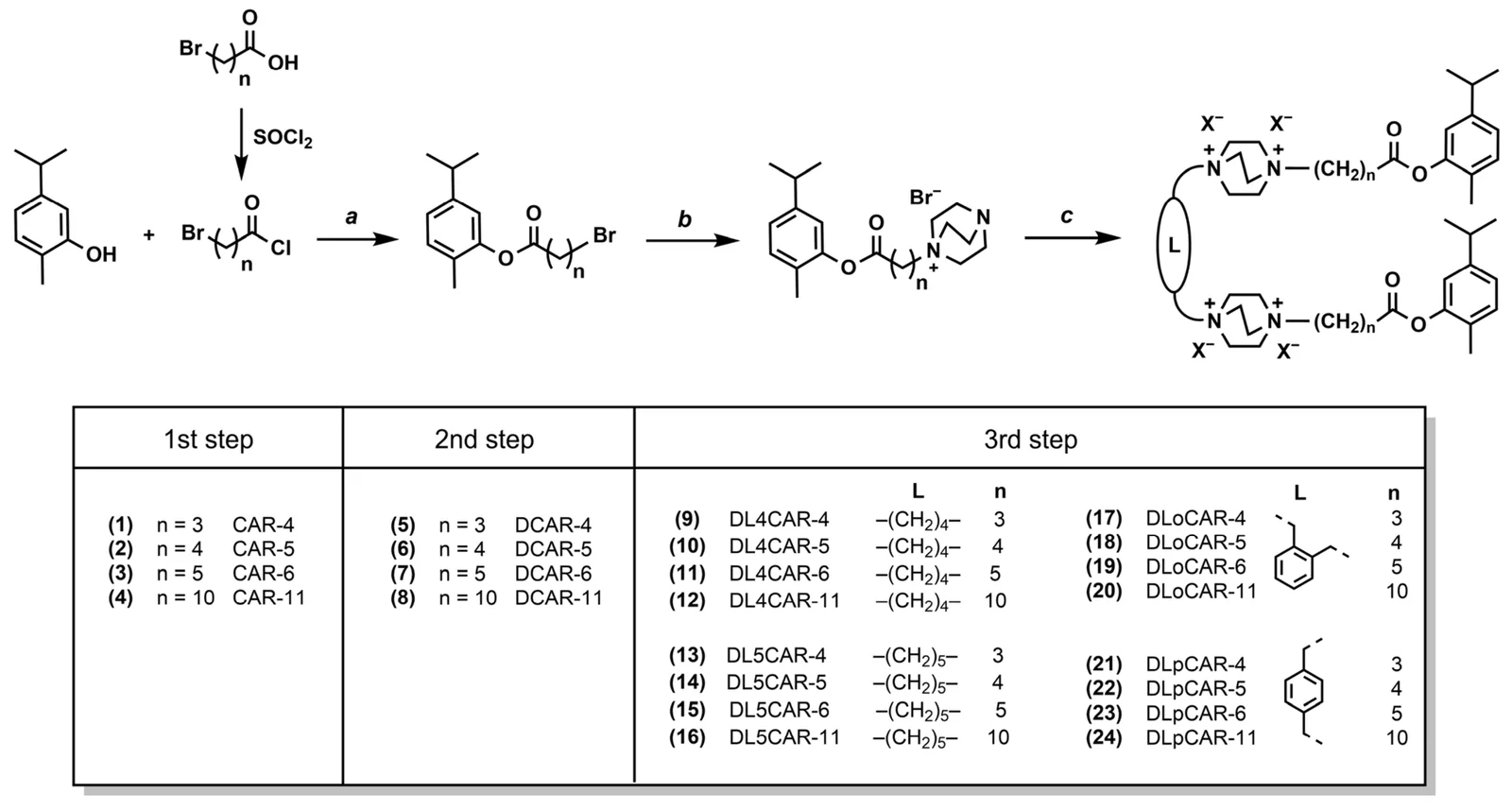
Synthesis scheme of the carvacrol–DABCO hybrids. Conditions: a—Et_3_N, DCM, RT, 16 h; b—DABCO, chloroform, reflux, 24 h; c—1,*ω*-dibromoalkane or *α*,*α*′-dihaloxylene, MeCN, 60 °C, 48 h; X = Br, Cl.

The general synthetic route to the target compounds is shown in Figure 2. For clarity, each compound was assigned both a sequential number and an abbreviated name reflecting its structural features.

The carvacrol hydroxyl group represents the most suitable site for modification. It has been previously shown that acylation of the hydroxyl group does not result in carvacrol losing its antibacterial activity [25]. Previously, we demonstrated that antibacterial activity exhibits a bell-shaped dependence on the length of the alkyl chain. The highest antibacterial activity was observed for compounds containing alkyl chains with 10–12 carbon atoms [16]. The calculated hydrophobicity values (ClogP) for these compounds, which contain a central linker composed of four methylene units, range from 3.28 to 5.39. In general, these values correspond to Lipinski’s rule, according to which the ClogP value should be less than 5 [26]. To achieve similar hydrophobic parameters, lateral linkers consisting of 3–5 methylene groups (ClogP: 2.74–6.54), as well as an extended linker with 10 methylene units, were selected. The latter was chosen to preserve a sufficiently long hydrophobic fragment in case of potential cleavage of carvacrol from the structure.

Earlier studies have shown that the RNA-hydrolyzing activity of tetracationic amphiphiles based on DABCO reaches its maximum when the central linker contains 4–6 carbon atoms [22,27]. To evaluate the influence of the central linker’s structure, particularly its length and rigidity, four different linkers were used, derived from 1,4-dibromobutane, 1,5-dibromopentane, *α*,*α*′-dibromo-*p*-xylene, and *α*,*α*′-dichloro-*o*-xylene.

The compounds were prepared following a previously reported synthetic methodology [28]. In the first step, carvacrol was acylated using the corresponding bromoacyl chlorides, prepared in situ from the corresponding ω-bromocarboxylic acids with thionyl chloride (Figure 2). In the second step, alkylation of DABCO was carried out in refluxing chloroform for 24 h, affording compounds **5**–**8** in high yields. In the third step, subsequent alkylation of compounds **5**–**8** with appropriate 1,*ω*-dibromoalkanes or *α*,*α*′-dihaloxylenes in acetonitrile at 60 °C for 48 h afforded the target compounds **9**–**24**.

As a result, four groups of cationic amphiphiles were synthesized, each comprising four compounds, varying in both the central and lateral linkers. The structures of all compounds were confirmed by ^1^H- and ^13^C-NMR spectroscopy, and mass spectrometry for second-step products (for full spectra see Supplementary Materials).

### 2.2. Antibacterial Activity

To evaluate antibacterial activity, one Gram-positive strain (*S. aureus*) and five Gram-negative strains (*E. coli*, *S. enterica*, *C. freundii*, *P. aeruginosa*, *A. baumannii*) were selected. Susceptibility testing was performed via serial dilution in 96-well microtiter plates. Carvacrol, ciprofloxacin hydrochloride, one of the most active previously reported cationic amphiphiles without additional pharmacophoric groups (DL4-12, see Figure 1A) [18], and the intermediate monocationic amphiphile DCAR-5 (compound **6**) were used as reference compounds. Minimum inhibitory concentration (MIC) values are reported in Table 1. The MIC values of ciprofloxacin, DL4-12, compounds **11**, **15**, **19**, and **23** against *S. aureus* correspond to previously reported data [28]. The MIC values of all substances presented in Table 1 were determined in parallel to ensure accurate comparison under identical test conditions.

As expected, based on the structural differences in bacterial cell walls, the Gram-positive *S. aureus* was the most susceptible to all tested compounds. MIC values for all compounds against this strain ranged from 4 to 31 µM. MIC values against Gram-negative strains ranged from 16 to 1000 µM.

The antibacterial activity of dimeric compounds **9**–**24** against all tested bacterial strains was significantly higher than that of compound **6**, further confirming the advantage of the dimeric scaffold bearing two DABCO moieties. Among them, compound **11** exhibited the same level of activity as the cationic amphiphile DL4-12 against four strains, showed eightfold higher activity against *P. aeruginosa*, but demonstrated 31-fold lower activity against *A. baumannii*.

Against all tested strains except *A. baumannii*, a bell-shaped relationship was observed between the antibacterial activity and the lateral linker length. In most cases, hybrids with *n* = 5 exhibited lower MIC values compared to those with *n* = 3 and 10.

The relatively high MIC values observed against *A. baumannii* may be attributed to its ability to form pericellular capsules [29], which can impede the action of cationic amphiphiles by screening the net negative charge of the bacterial cell surface from cationic agents [30]. Given that DL4-12 demonstrated high activity against *A. baumannii*, and an increase in potency was observed among the carvacrol-based hybrids **9**–**24** against this strain as the lateral linker length increased, it can be hypothesized that more hydrophobic lateral substituents are required for the effective inhibition of *A. baumannii* by cationic amphiphiles.

Hybrids featuring the central *o*-xylylene linker demonstrated a lower level of antibacterial activity, whereas those containing tetramethylene, pentamethylene, and *p*-xylylene linkers displayed roughly comparable potencies.

**Figure 3:**
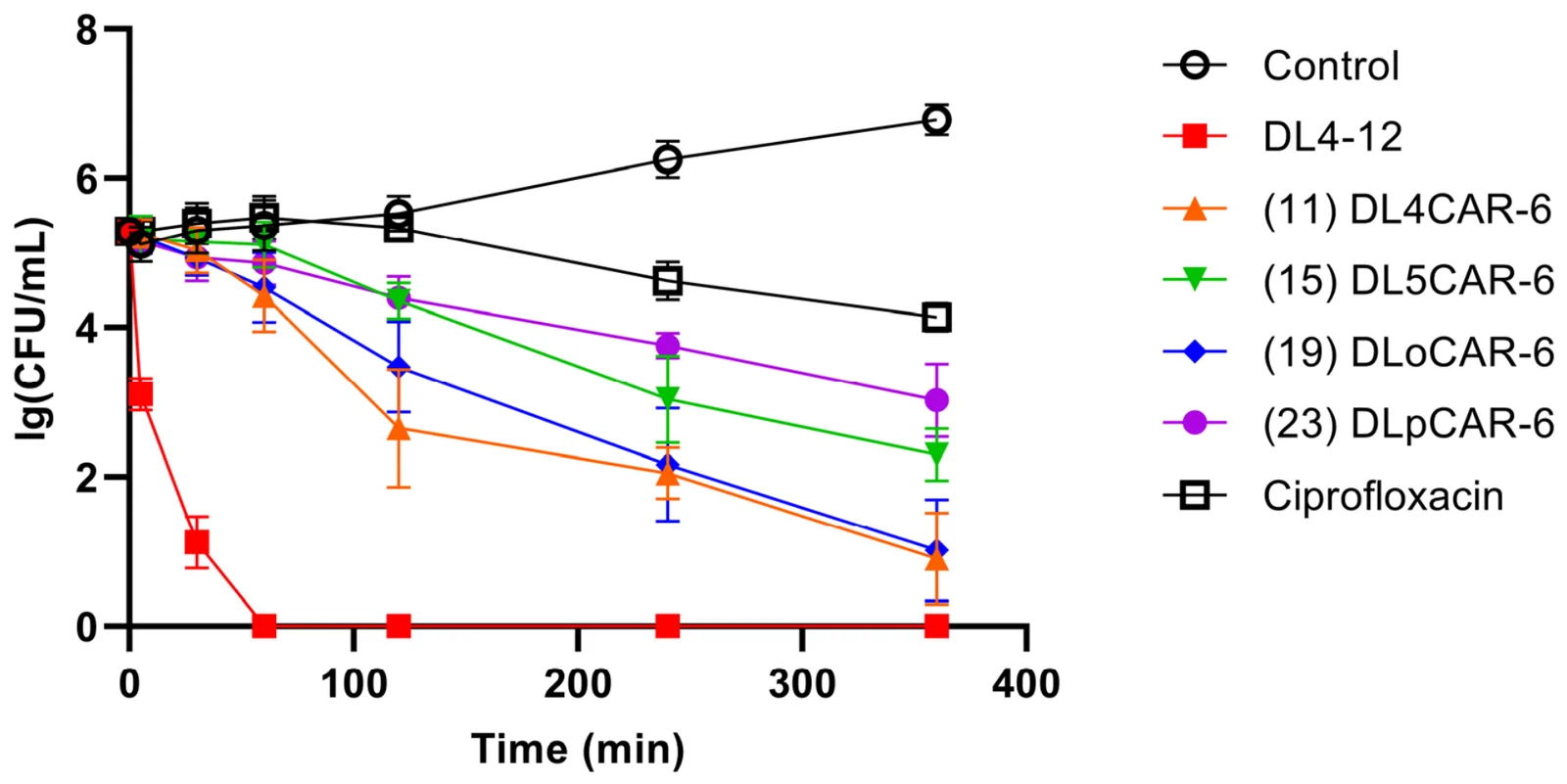
Time-kill kinetics against *S. aureus*. Compounds were tested at concentrations: DL4-12, DL4CAR-6—4 µM, DL5CAR-6, DLoCAR-6, DLpCAR-6—8 µM, and ciprofloxacin—2 µM. Data are presented as mean ± SD.

An important characteristic that indirectly suggests the mechanism of antibacterial activity is the eradication rate of infectious agents. For compounds **11**, **15**, **19**, **23**, DL4-12, and ciprofloxacin, the time-kill kinetics against *S. aureus* were investigated. Measurements were performed at concentrations corresponding to each compound’s *S. aureus* MIC (Table 1). As seen from the obtained results (Figure 3), the cationic amphiphiles begin to inhibit *S. aureus* faster than ciprofloxacin. Specifically, after 6 h of incubation, the population of surviving bacteria treated with the synthesized compounds dropped to 10^1^–10^3^ CFU/mL, demonstrating higher efficacy in bacterial population reduction compared to the reference drug (10^4^ CFU/mL). Such a faster antimicrobial onset is likely attributed to significant damage to the bacterial cell wall, which has been previously confirmed for these compounds using transmission electron microscopy [28]. This is most clearly demonstrated by the cationic amphiphile DL4-12.

### 2.3. RNA-Hydrolyzing Activity

**Figure 4:**
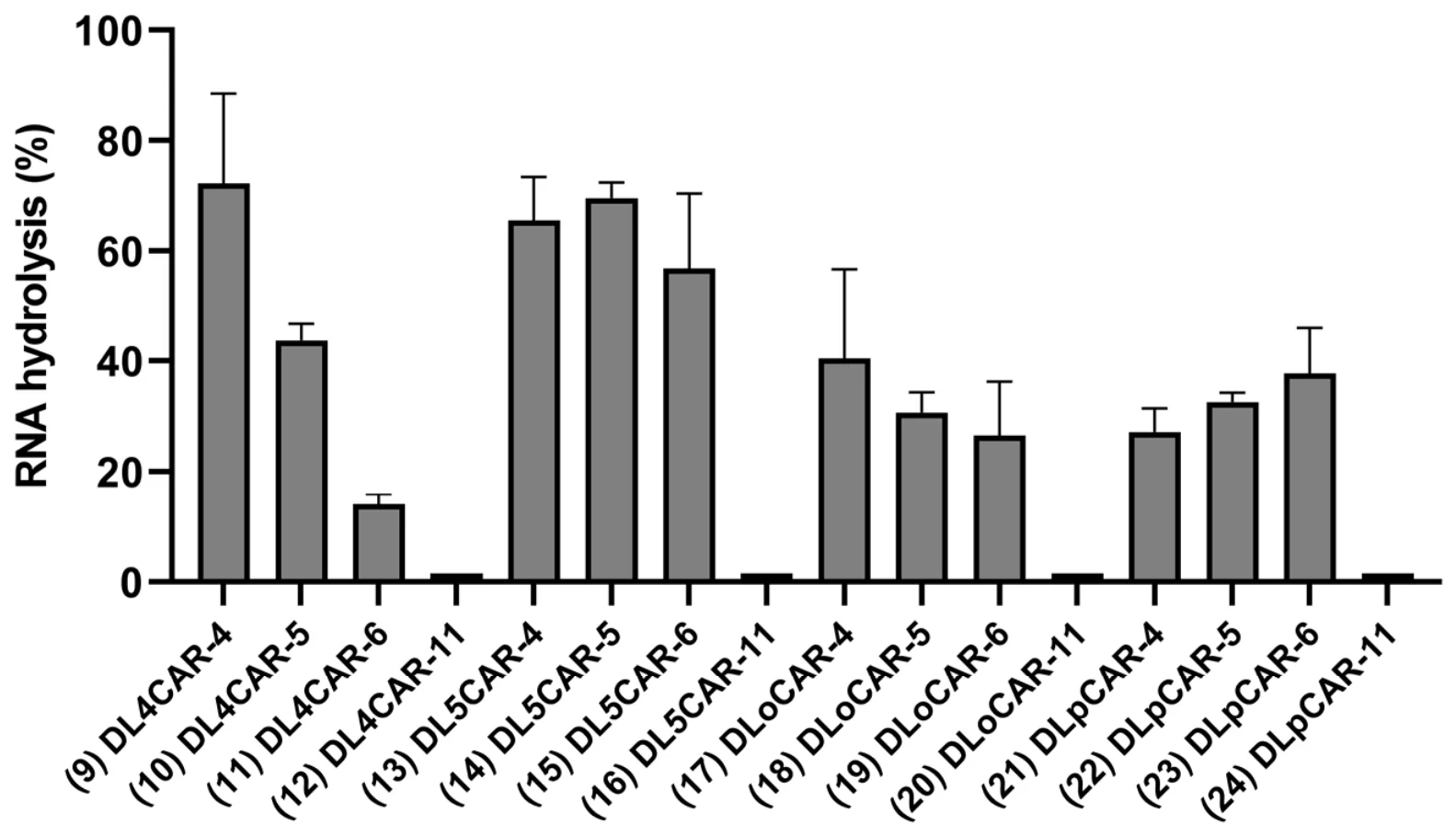
Cleavage efficiency of the model ribooligonucleotide by compounds **9**–**24**. Data are presented as mean ± SD.

It was previously hypothesized, and subsequently supported by experimental data [21], that bacterial RNA represents a potential target for the cationic amphiphile DL4-12. Therefore RNA-hydrolyzing activity of the synthesized compounds was evaluated. A fluorescently labeled 17-mer ribooligonucleotide (5′-AGUUACCUCGGAUGCAA-3′-FAM) was used as a model substrate. Figure 4 presents data on the cleavage of the model ribooligonucleotide.

As evident from the data, all compounds containing the longest lateral linker (14.9 Å, with 10 methylene units) showed negligible RNA cleavage. All other compounds exhibited pronounced RNA-hydrolyzing activity; however, a clear correlation between the extent of cleavage and the length of the lateral linker was only observed within the group of compounds featuring a central linker composed of four methylene units (compounds **9**–**12**).

Compounds with a central pentamethylene linker (7.4 Å) generally exhibited higher RNA hydrolysis levels compared to those with shorter tetramethylene (6.4 Å) and *o*-xylylene (5.4 Å) linkers, as well as the longer *p*-xylylene linker (7.8 Å). Exceptions were compounds **9** and **13**, which showed comparable hydrolysis efficiency.

No clear correlation between antibacterial and RNA-hydrolyzing activities was observed. Nevertheless, compounds lacking RNA-hydrolyzing activity (compounds **12**, **16**, **20**, **24**) generally showed reduced antibacterial efficacy across all bacterial strains within their respective groups. Thus, it can be concluded that RNA cleavage may contribute to antibacterial activity.

### 2.4. Hemolytic Activity

One of the major limitations in the application of cationic amphiphile-based antibacterial agents is their high hemolytic toxicity. Typically, amphiphiles exhibiting strong antibacterial activity also show high hemolytic activity, primarily due to the dominant mechanism of action involving membrane disruption.

Hemolytic activity of the synthesized compounds was assessed over a concentration range where significant antibacterial activity was observed. Since compounds **12**, **16**, **20**, and **24** (with *n* = 10) exhibited relatively high MIC values and lacked RNA-hydrolyzing activity, they were excluded from further evaluation.

Hemolysis experiments revealed that the majority of the tested compounds induced less than 10% hemolysis even at the maximum concentration of 500 µM (Table 2). An exception is the reference compound DL4-12, whose hemolytic activity increases sharply from 0.392 ± 0.013% at a concentration of 2 µM to 74.2 ± 2.6% at a concentration of 32 µM.

All compounds with a central tetramethylene linker (6.4 Å) showed higher hemolysis levels compared to those with shorter (5.4 Å) or longer (7.4 Å and 7.8 Å) linkers, indicating a bell-shaped relationship. This trend was most apparent at the highest tested concentration (500 µM). The lowest hemolysis levels were observed for the longest central linker (7.8 Å).

The correlation between hemolytic activity and the length of the lateral linker among compounds with a four-methylene central linker (compounds **9**–**11**) was nonlinear. Compound **10**, containing four methylene groups in the lateral chain, showed lower hemolytic activity compared to compounds **9** and **11**, which contained three and five methylene units, respectively. For other compounds, this effect was less pronounced, likely due to their overall lower hemolytic activity. Nevertheless, for all compounds, the highest level of hemolysis was observed when the lateral linker length was 8.7 Å.

The correlation between hemolytic activity and the length of the lateral linker among compounds with a four-methylene central linker (compounds **9**–**11**) was nonlinear. Compound **10**, containing four methylene groups in the lateral chain, showed lower hemolytic activity compared to compounds **9** and **11**, which contained three and five methylene units, respectively. For other compounds, this effect was less pronounced, likely due to their overall lower hemolytic activity.

In general, hemolytic activity of compounds with flexible and rigid central linkers was comparable. However, compounds containing a *p*-xylylene central linker consistently showed significantly lower hemolytic activity regardless of the lateral linker structure. Importantly, this low hemolytic activity was maintained even at the highest concentrations tested.

Compound **11**, which showed the highest antibacterial activity against all tested bacterial strains, exhibited the greatest hemolytic toxicity at high concentrations (125–500 µM). However, within the range of MICs (4–16 µM), this compound caused negligible erythrocyte lysis (hemolysis < 1.37%).

### 2.5. Cytotoxicity

The most active synthesized compounds DL4CAR-6, DL5CAR-6, and compound DL4-12 were evaluated for their in vitro antiproliferative effect in normal and cancer cell lines. Each compound was tested on RPMI8226, LMTK cell lines and MEF primary cells (Table 3). Compound DL4CAR-6 showed low cytotoxicity against all cell lines (IC_50_ ranges from 114 to 250 µM). The cytotoxic doses of all compounds were higher than their effective doses against bacterial strains.

Based on the obtained results, the incorporation of carvacrol into the structure of tetracationic amphiphiles significantly decreased cytotoxicity, thereby improving the selectivity. Given that carvacrol itself is a relatively small hydrophobic molecule (log P ~ 3.5) that non-specifically partitions into lipid bilayers and induces membrane depolarization in both prokaryotes and eukaryotes [31,32], this enhancement in selectivity is presumably attributable to the effect of carvacrol moieties on the physicochemical parameters of the amphiphiles, including the hydrophilic–lipophilic balance, polarizability, and spatial configuration.

## 3. Materials and Methods

### 3.1. Chemical Synthesis

The following reagents were used in this study: 1,4-diazabicyclo[2.2.2]octane, 1,5-dibromopentane, 4-bromobutyric acid, 5-bromovaleric acid, 5-isopropyl-2-methylphenol (carvacrol), 6-bromohexanoic acid, *α*,*α*′-dibromo-*p*-xylene, *α*,*α*′-dichloro-*o*-xylene (Sigma-Aldrich, St. Louis, MO, USA), 1,4-dibromobutane (Alfa Aesar, Heysham, UK), 11-bromoundecanoic acid (Acros Organics, Loughborough, UK), and thionyl chloride (Fluka, Buchs, Switzerland). All solvents were purchased from Reachem (Moscow, Russia); deuterated solvents were purchased from Astrachem (Moscow, Russia). Organic solvents were dried and purified by standard procedures if necessary.

TLC was carried out on Silica gel 60 F_254_ plates (Merck, Darmstadt, Germany) using appropriate solvent systems; spots were visualized under UV irradiation.

NMR spectra were acquired on Avance 300, 400, 600 and DRX 500 instruments (Bruker, Ettlingen, Germany) in appropriate deuterated solvents at 30 °C. Chemical shifts (δ) are reported in ppm and referenced to residual solvent signals (CDCl_3_: 7.26 and 77.16 ppm, DMSO-d6: 2.50 and 39.52 ppm for ^1^H and ^13^C NMR respectively). Coupling constants (*J*) are reported in Hz.

ESI mass spectra were recorded on an Agilent ESI MSD XCT Ion Trap (Agilent Technologies, Santa Clara, CA, USA) in positive mode.

The synthesis of compounds **3**, **7**, **11**, **15**, **19**, and **23** was described previously [28].

#### 3.1.1. General Procedure for the Synthesis of Compounds **1**–**4**

To *ω*-bromocarboxylic acid (2.56 mmol), thionyl chloride (1.30 mL, 17.9 mmol) was added. The reaction mixture was stirred at 50 °C for 3 h. The excess thionyl chloride was removed under reduced pressure. The resulting acyl chloride residue was dissolved in DCM (5.0 mL), and the mixture of carvacrol (347 mg, 2.31 mmol) and triethylamine (1.07 mL, 7.69 mmol) was added dropwise at 0 °C. The reaction mixture was then allowed to warm to room temperature and stirred. The progress of the reaction was monitored by TLC (DCM/hexane 1:1). Upon completion (16 h), the reaction mixture was diluted with DCM (40 mL), washed sequentially with water (2 × 40 mL) and brine (40 mL). The organic layer was dried over Na_2_SO_4_ (anh.) and filtered. The solvent was removed under reduced pressure, and the residual crude product was dried under vacuum.

5-Isopropyl-2-methylphenyl 4-bromobutanoate (**1**, CAR-4). Colorless oil, 475 mg, 66% yield. R_f_ 0.30 (hexane/DCM 1:1). ^1^H NMR (500 MHz, CDCl_3_) δ 7.15 (d, *J* = 7.9 Hz, 1H), 7.03 (dd, *J* = 7.9, 1.9 Hz, 1H), 6.86 (d, *J* = 1.8 Hz, 1H), 3.56 (t, *J* = 6.4 Hz, 2H), 2.88 (hept, *J* = 6.8 Hz, 1H), 2.81 (t, *J* = 7.2 Hz, 2H), 2.31 (p, *J* = 6.8 Hz, 2H), 2.14 (s, 3H), 1.23 (d, *J* = 6.9 Hz, 6H). ^13^C NMR (126 MHz, CDCl_3_) δ 171.03, 149.18, 148.25, 131.05, 127.17, 124.36, 119.80, 33.68, 32.76, 32.43, 27.77, 24.04, 15.97.

5-Isopropyl-2-methylphenyl 5-bromopentanoate (**2**, CAR-5). Colorless oil, 692 mg, 96% yield. R_f_ 0.34 (hexane/DCM 1:1). ^1^H NMR (500 MHz, CDCl_3_) δ 7.15 (d, *J* = 7.8 Hz, 1H), 7.02 (dd, *J* = 7.8, 1.8 Hz, 1H), 6.85 (d, *J* = 1.8 Hz, 1H), 3.47 (t, *J* = 6.4 Hz, 2H), 2.87 (hept, *J* = 6.9 Hz, 1H), 2.63 (t, *J* = 7.2 Hz, 2H), 2.13 (s, 3H), 2.06–1.89 (m, 4H), 1.23 (d, *J* = 6.9 Hz, 6H). ^13^C NMR (126 MHz, CDCl_3_) δ 171.54, 149.24, 148.22, 131.02, 127.17, 124.28, 119.83, 33.67, 33.36, 33.13, 32.10, 24.03, 23.69, 15.99.

5-Isopropyl-2-methylphenyl 11-bromoundecanoate (**4**, CAR-11). Colorless oil, 759 mg, 83% yield. R_f_ 0.57 (hexane/DCM 1:1). ^1^H NMR (400 MHz, CDCl_3_) δ 7.14 (d, *J* = 7.8 Hz, 1H), 7.01 (dd, *J* = 7.8, 1.8 Hz, 1H), 6.85 (d, *J* = 1.8 Hz, 1H), 3.41 (t, *J* = 6.9 Hz, 2H), 2.87 (hept, *J* = 6.9 Hz, 1H), 2.58 (t, *J* = 7.5 Hz, 2H), 2.13 (s, 3H), 1.87 (h, *J* = 9.0 Hz, 2H), 1.78 (p, *J* = 7.5 Hz, 2H), 1.49–1.37 (m, 4H), 1.38–1.28 (m, 8H), 1.23 (d, *J* = 6.9 Hz, 6H). ^13^C NMR (126 MHz, CDCl_3_) δ 172.24, 149.34, 148.13, 130.98, 127.25, 124.16, 119.89, 34.40, 34.25, 33.67, 32.92, 29.49, 29.46, 29.34, 29.29, 28.86, 28.27, 25.18, 24.04, 15.98.

#### 3.1.2. General Procedure for the Synthesis of Compounds **5**–**8**

A solution of compound **1**–**4** (1.56 mmol) in chloroform (2.0 mL) was added to a solution of DABCO (350 mg, 3.12 mmol) in chloroform (3.0 mL), and the reaction mixture was refluxed. The progress of the reaction was monitored by TLC (DCM/hexane 1:1). Upon completion (24 h), the solvent was removed under reduced pressure. The residue was redissolved in methanol (2.0 mL) and added dropwise to diethyl ether (40 mL). The resulting precipitate was filtered off, washed with diethyl ether and dried under high vacuum.

1-(4-(5-Isopropyl-2-methylphenoxy)-4-oxobutyl)-1,4-diazabicyclo[2.2.2]octan-1-ium bromide (**5**, DCAR-4). Pale yellow solid, 520 mg, 81% yield. ^1^H NMR (300 MHz, DMSO-d6) δ 7.20 (d, *J* = 7.8 Hz, 1H), 7.05 (dd, *J* = 7.7, 1.8 Hz, 1H), 6.93 (d, *J* = 1.8 Hz, 1H), 3.40–3.26 (m, 8H), 3.03 (t, *J* = 7.3 Hz, 6H), 2.86 (hept, *J* = 6.9 Hz, 1H), 2.74 (t, *J* = 7.2 Hz, 2H), 2.07 (s, 3H), 2.14–1.98 (m, 2H), 1.17 (d, *J* = 6.9 Hz, 6H). ^13^C NMR (126 MHz, DMSO-d6) δ 170.42, 148.87, 147.53, 130.70, 126.81, 123.89, 119.62, 62.13, 51.53, 44.66, 32.76, 30.06, 23.71, 16.88, 15.36. MS ESI (*m*/*z*): [M − Br]^+^ expected for M = C_20_H_31_BrN_2_O_2_ 331.2, found 331.4.

1-(5-(5-Isopropyl-2-methylphenoxy)-5-oxopentyl)-1,4-diazabicyclo[2.2.2]octan-1-ium bromide (**6**, DCAR-5). Pale yellow solid, 611 mg, 92% yield. ^1^H NMR (500 MHz, DMSO-d6) δ 7.19 (d, *J* = 7.8 Hz, 1H), 7.05 (dd, *J* = 7.8, 1.9 Hz, 1H), 6.90 (d, *J* = 1.8 Hz, 1H), 3.32–3.25 (m, 8H), 3.03 (t, *J* = 7.5 Hz, 6H), 2.85 (hept, *J* = 6.9 Hz, 1H), 2.70 (t, *J* = 7.3 Hz, 2H), 2.06 (s, 3H), 1.84–1.74 (m, 2H), 1.73–1.63 (m, 2H), 1.17 (d, *J* = 6.9 Hz, 6H). ^13^C NMR (126 MHz, DMSO-d6) δ 171.39, 149.27, 147.90, 131.06, 127.18, 124.19, 120.03, 62.99, 51.79, 44.98, 33.13, 32.98, 24.10, 21.74, 20.85, 15.74. MS ESI (*m*/*z*): [M − Br]^+^ expected for M = C_21_H_33_BrN_2_O_2_ 345.3, found 345.4.

1-(11-(5-isopropyl-2-methylphenoxy)-11-oxoundecyl)-1,4-diazabicyclo[2.2.2]octan-1-ium bromide (**8**, DCAR-11). White solid, 626 mg, 95% yield. ^1^H NMR (400 MHz, DMSO-d6) δ 7.18 (d, *J* = 7.8 Hz, 1H), 7.04 (dd, *J* = 7.8, 1.9 Hz, 1H), 6.88 (d, *J* = 1.8 Hz, 1H), 3.25 (t, *J* = 7.4 Hz, 6H), 3.21–3.12 (m, 2H), 3.00 (t, *J* = 7.4 Hz, 6H), 2.85 (hept, *J* = 6.9 Hz, 1H), 2.59 (t, *J* = 7.3 Hz, 2H), 2.04 (s, 3H), 1.71–1.58 (m, 4H), 1.41–1.24 (m, 12H), 1.16 (d, *J* = 6.9 Hz, 6H). ^13^C NMR (126 MHz, DMSO-d6) δ 171.55, 149.09, 147.63, 130.77, 126.92, 123.86, 119.75, 63.16, 51.46, 44.71, 33.36, 32.86, 28.82, 28.77, 28.69, 28.57, 28.48, 25.91, 24.47, 23.83, 21.07, 15.41. MS ESI (*m*/*z*): [M − Br]^+^ expected for M = C_27_H_45_BrN_2_O_2_ 429.4, found 429.7.

#### 3.1.3. General Procedure for the Synthesis of Compounds **9**–**24**

To a solution of compound **5**–**8** (1.20 mmol) in acetonitrile (4.0 mL), a dihalohydrocarbon (0.30 mmol) was added. The reaction mixture was stirred at 60 °C for 24 h. A second portion of the dihalohydrocarbon (0.30 mmol) was then added, and stirring was continued at 60 °C for an additional 24 h. The reaction mixture was allowed to cool to room temperature, and acetone (16 mL) was added to induce precipitation. The resulting precipitate was collected by filtration, washed with acetonitrile (20 mL) and acetone (2 × 20 mL), and dried under vacuum.

4,4′-(Butane-1,4-diyl)bis(1-(4-(5-isopropyl-2-methylphenoxy)-4-oxobutyl)-1,4-diazabicyclo[2.2.2]octane-1,4-diium) tetrabromide (**9**, DL4CAR-4). Prepared from compound **5** and 1,4-dibromobutane. Pale yellow solid, 75% yield. ^1^H NMR (500 MHz, DMSO-d6) δ 7.20 (d, *J* = 7.8 Hz, 2H), 7.06 (d, *J* = 7.8 Hz, 2H), 6.94 (s, 2H), 4.01 (s, 24H), 3.75–3.64 (m, 8H), 2.86 (hept, *J* = 7.0 Hz, 2H), 2.78 (t, *J* = 7.2 Hz, 4H), 2.17–2.09 (m, 4H), 2.08 (s, 6H), 1.87–1.80 (m, 4H), 1.17 (d, *J* = 6.9 Hz, 12H). ^13^C NMR (126 MHz, DMSO-d6) δ 170.31, 148.85, 147.54, 130.71, 126.79, 123.94, 119.60, 62.36, 50.52, 50.45, 32.76, 29.75, 23.72, 18.54, 17.22, 15.36.

4,4′-(Butane-1,4-diyl)bis(1-(5-(5-isopropyl-2-methylphenoxy)-5-oxopentyl)-1,4-diazabicyclo[2.2.2]octane-1,4-diium) tetrabromide (**10**, DL4CAR-5). Prepared from compound **6** and 1,4-dibromobutane. Pale yellow solid, 81% yield. ^1^H NMR (400 MHz, DMSO-d6) δ 7.19 (d, *J* = 7.8 Hz, 2H), 7.05 (dd, *J* = 7.8, 1.8 Hz, 2H), 6.92 (d, *J* = 1.8 Hz, 2H), 4.05–3.91 (m, 24H), 3.74–3.62 (m, 8H), 2.85 (hept, *J* = 6.9 Hz, 2H), 2.72 (t, *J* = 7.3 Hz, 4H), 2.06 (s, 6H), 1.91–1.77 (m, 8H), 1.76–1.64 (m, 4H), 1.17 (d, *J* = 6.9 Hz, 12H). ^13^C NMR (101 MHz, DMSO-d6) δ 171.01, 148.94, 147.59, 130.74, 126.85, 123.87, 119.73, 62.91, 62.34, 50.49, 50.42, 32.81, 32.57, 23.78, 21.12, 20.85, 18.59, 15.44.

4,4′-(Butane-1,4-diyl)bis(1-(11-(5-isopropyl-2-methylphenoxy)-11-oxoundecyl)-1,4-diazabicyclo[2.2.2]octane-1,4-diium) tetrabromide (**12**, DL4CAR-11). Prepared from compound **8** and 1,4-dibromobutane. White solid, 74% yield. ^1^H NMR (400 MHz, DMSO-d6) δ 7.18 (d, *J* = 7.8 Hz, 2H), 7.04 (dd, *J* = 7.8, 1.8 Hz, 2H), 6.88 (d, *J* = 1.8 Hz, 2H), 4.03–3.83 (m, 24H), 3.72–3.61 (m, 4H), 3.58–3.50 (m, 4H), 2.85 (hept, *J* = 7.0 Hz, 2H), 2.59 (t, *J* = 7.3 Hz, 4H), 2.04 (s, 6H), 1.79 (s, 4H), 1.73–1.60 (m, 8H), 1.43–1.25 (m, 26H), 1.17 (d, *J* = 6.9 Hz, 12H). ^13^C NMR (126 MHz, DMSO-d6) δ 171.51, 149.05, 147.61, 130.74, 126.85, 123.82, 119.69, 63.50, 62.45, 50.56, 50.41, 33.35, 32.80, 28.76, 28.70, 28.64, 28.45, 25.53, 24.43, 23.77, 21.35, 18.57, 15.34.

4,4′-(Pentane-1,5-diyl)bis(1-(4-(5-isopropyl-2-methylphenoxy)-4-oxobutyl)-1,4-diazabicyclo[2.2.2]octane-1,4-diium) tetrabromide (**13**, DL5CAR-4). Prepared from compound **5** and 1,5-dibromopentane. Pale yellow solid, 72% yield. ^1^H NMR (500 MHz, DMSO-d6) δ 7.20 (d, *J* = 7.8 Hz, 2H), 7.06 (d, *J* = 7.8 Hz, 2H), 6.94 (d, *J* = 2.0 Hz, 2H), 4.01 (s, 24H), 3.73–3.63 (m, 8H), 2.86 (hept, *J* = 7.0 Hz, 2H), 2.77 (t, *J* = 7.2 Hz, 4H), 2.17–2.07 (m, 4H), 2.08 (s, 6H), 1.89–1.79 (m, 4H), 1.43–1.33 (m, 2H), 1.17 (d, *J* = 6.9 Hz, 12H). ^13^C NMR (126 MHz, DMSO-d6) δ 170.63, 149.17, 147.87, 131.04, 127.12, 124.26, 119.93, 63.13, 62.68, 50.77, 33.09, 30.09, 24.05, 22.60, 21.10, 17.54, 15.69.

4,4′-(Pentane-1,5-diyl)bis(1-(5-(5-isopropyl-2-methylphenoxy)-5-oxopentyl)-1,4-diazabicyclo[2.2.2]octane-1,4-diium) tetrabromide (**14**, DL5CAR-5). Prepared from compound **6** and 1,5-dibromopentane. Pale yellow solid, 86% yield. ^1^H NMR (500 MHz, DMSO-d6) δ 7.19 (d, *J* = 7.8 Hz, 2H), 7.05 (dd, *J* = 7.8, 1.8 Hz, 2H), 6.92 (d, *J* = 1.7 Hz, 2H), 4.04–3.92 (m, 24H), 3.66 (q, *J* = 9.2 Hz, 8H), 2.85 (hept, *J* = 6.6 Hz, 2H), 2.72 (t, *J* = 7.3 Hz, 4H), 2.06 (s, 6H), 1.89–1.76 (m, 8H), 1.69 (p, *J* = 7.8 Hz, 4H), 1.41–1.31 (m, 2H), 1.16 (d, *J* = 6.9 Hz, 12H). ^13^C NMR (126 MHz, DMSO-d6) δ 171.06, 148.97, 147.63, 130.78, 126.89, 123.93, 119.77, 62.90, 62.80, 50.45, 50.41, 32.86, 32.60, 30.76, 23.83, 22.34, 21.15, 20.86, 15.48.

4,4′-(Pentane-1,5-diyl)bis(1-(11-(5-isopropyl-2-methylphenoxy)-11-oxoundecyl)-1,4-diazabicyclo[2.2.2]octane-1,4-diium) tetrabromide (**16**, DL5CAR-11). Prepared from compound **8** and 1,5-dibromopentane. Pale yellow solid, 76% yield. ^1^H NMR (400 MHz, DMSO-d6) δ 7.18 (d, *J* = 7.8 Hz, 2H), 7.04 (dd, *J* = 7.8, 1.8 Hz, 2H), 6.88 (d, *J* = 1.8 Hz, 2H), 3.99–3.86 (m, 24H), 3.67–3.59 (m, 4H), 3.57–3.49 (m, 4H), 2.85 (hept, *J* = 6.9 Hz, 2H), 2.59 (t, *J* = 7.3 Hz, 4H), 2.04 (s, 6H), 1.81 (dt, *J* = 16.0, 7.2 Hz, 4H), 1.76–1.59 (m, 8H), 1.42–1.24 (m, 26H), 1.17 (d, *J* = 6.9 Hz, 12H). ^13^C NMR (126 MHz, DMSO-d6) δ 171.22, 148.92, 147.40, 130.53, 126.65, 123.56, 119.50, 63.36, 62.80, 50.36, 50.26, 33.22, 32.61, 28.56, 28.49, 28.43, 28.27, 28.22, 25.39, 24.26, 23.55, 22.20, 21.18, 20.66, 15.14.

4,4′-(1,2-Phenylenebis(methylene))bis(1-(4-(5-isopropyl-2-methylphenoxy)-4-oxobutyl)-1,4-diazabicyclo[2.2.2]octane-1,4-diium) dibromide dichloride (**17**, DLoCAR-4). Prepared from compound **5** and *α*,*α*′-dichloro-*o*-xylene. Pale yellow solid, 86% yield. ^1^H NMR (400 MHz, DMSO-d6) δ 7.85 (dd, *J* = 5.8, 3.5 Hz, 2H), 7.75 (dd, *J* = 5.8, 3.3 Hz, 2H), 7.15 (d, *J* = 7.9 Hz, 2H), 7.02 (dd, *J* = 7.8, 1.8 Hz, 2H), 6.89 (d, *J* = 1.8 Hz, 2H), 5.45 (s, 4H), 4.18 (t, *J* = 7.3 Hz, 12H), 3.92 (t, *J* = 7.4 Hz, 12H), 3.61–3.52 (m, 4H), 2.81 (hept, *J* = 6.9 Hz, 2H), 2.68 (t, *J* = 7.2 Hz, 4H), 2.10–1.99 (m, 10H), 1.16 (d, *J* = 6.9 Hz, 12H). ^13^C NMR (126 MHz, DMSO-d6) δ 170.25, 148.84, 147.54, 135.67, 131.48, 130.71, 128.45, 126.79, 123.94, 119.61, 62.30, 62.14, 60.38, 50.39, 49.55, 32.78, 29.79, 23.72, 17.12, 15.35.

4,4′-(1,2-Phenylenebis(methylene))bis(1-(5-(5-isopropyl-2-methylphenoxy)-5-oxopentyl)-1,4-diazabicyclo[2.2.2]octane-1,4-diium) dibromide dichloride (**18**, DLoCAR-5). Prepared from compound **6** and *α*,*α*′-dichloro-*o*-xylene. Pale yellow solid, 93% yield. ^1^H NMR (400 MHz, DMSO-d6) δ 7.88 (dd, *J* = 5.8, 3.6 Hz, 2H), 7.79 (dd, *J* = 5.8, 3.4 Hz, 2H), 7.22–7.15 (m, 2H), 7.05 (dd, *J* = 7.8, 1.9 Hz, 2H), 6.90 (d, *J* = 1.8 Hz, 2H), 5.42 (s, 4H), 4.17 (d, *J* = 7.7 Hz, 12H), 3.88 (d, *J* = 7.2 Hz, 12H), 3.59 (t, *J* = 7.8 Hz, 4H), 2.84 (hept, *J* = 6.9 Hz, 2H), 2.70 (t, *J* = 7.3 Hz, 4H), 2.05 (s, 6H), 1.79–1.75 (m, 4H), 1.66 (q, *J* = 7.4 Hz, 4H), 1.16 (d, *J* = 6.9 Hz, 12H). ^13^C NMR (101 MHz, DMSO-d6) δ 171.02, 148.97, 147.62, 135.73, 131.56, 130.77, 128.49, 128.41, 126.87, 123.91, 119.74, 62.88, 62.18, 50.39, 49.58, 32.85, 32.56, 23.82, 21.16, 20.75, 15.46.

4,4′-(1,2-Phenylenebis(methylene))bis(1-(11-(5-isopropyl-2-methylphenoxy)-11-oxoundecyl)-1,4-diazabicyclo[2.2.2]octane-1,4-diium) dibromide dichloride (**20**, DLoCAR-11). Prepared from compound **8** and *α*,*α*′-dichloro-*o*-xylene. Pale yellow solid, 82% yield. ^1^H NMR (600 MHz, DMSO-d6) δ 7.87 (dd, *J* = 5.7, 3.5 Hz, 2H), 7.78 (dd, *J* = 5.8, 3.4 Hz, 2H), 7.18 (d, *J* = 7.8 Hz, 2H), 7.04 (dd, *J* = 7.8, 1.8 Hz, 2H), 6.87 (d, *J* = 1.8 Hz, 2H), 5.42 (s, 4H), 4.17 (t, *J* = 7.5 Hz, 12H), 3.85 (t, *J* = 7.7 Hz, 12H), 3.50 (t, *J* = 8.5 Hz, 4H), 2.85 (hept, *J* = 6.9 Hz, 2H), 2.58 (t, *J* = 7.3 Hz, 4H), 2.04 (s, 6H), 1.64 (h, *J* = 6.2 Hz, 8H), 1.39–1.22 (m, 24H), 1.16 (d, *J* = 6.9 Hz, 12H). ^13^C NMR (151 MHz, DMSO-d6) δ 171.44, 149.02, 147.56, 135.67, 131.49, 130.69, 128.45, 126.83, 123.78, 119.67, 63.25, 62.11, 50.27, 49.51, 40.04, 39.90, 33.30, 32.78, 28.73, 28.65, 28.62, 28.42, 28.35, 25.51, 24.40, 23.75, 21.16, 15.32.

4,4′-(1,4-Phenylenebis(methylene))bis(1-(4-(5-isopropyl-2-methylphenoxy)-4-oxobutyl)-1,4-diazabicyclo[2.2.2]octane-1,4-diium) tetrabromide (**21**, DLpCAR-4). Prepared from compound **5** and *α*,*α*′-dibromo-*p*-xylene. Pale yellow solid, 90% yield. ^1^H NMR (400 MHz, DMSO-d6) δ 7.74 (s, 4H), 7.15 (d, *J* = 7.6 Hz, 2H), 7.01 (dd, *J* = 7.8, 1.9 Hz, 2H), 6.88 (d, *J* = 1.8 Hz, 2H), 4.99 (s, 4H), 4.04–3.92 (m, 24H), 3.58–3.48 (m, 4H), 2.81 (hept, *J* = 6.9 Hz, 2H), 2.71 (t, *J* = 7.2 Hz, 4H), 2.06–1.95 (m, 10H), 1.12 (d, *J* = 6.9 Hz, 12H). ^13^C NMR (126 MHz, DMSO-d6) δ 170.30, 148.83, 147.54, 133.71, 130.71, 128.99, 126.79, 123.94, 119.60, 65.55, 62.63, 50.54, 50.07, 32.77, 29.64, 23.72, 17.15, 15.36.

4,4′-(1,4-Phenylenebis(methylene))bis(1-(5-(5-isopropyl-2-methylphenoxy)-5-oxopentyl)-1,4-diazabicyclo[2.2.2]octane-1,4-diium) tetrabromide (**22**, DLpCAR-5). Prepared from compound **6** and *α*,*α*′-dibromo-*p*-xylene. Pale yellow solid, 93% yield. ^1^H NMR (400 MHz, DMSO-d6) δ 7.77 (s, 4H), 7.18 (dd, *J* = 7.7, 0.8 Hz, 2H), 7.04 (dd, *J* = 7.8, 1.9 Hz, 2H), 6.90 (d, *J* = 1.8 Hz, 2H), 5.01 (s, 4H), 4.03 (t, *J* = 7.1 Hz, 12H), 3.94 (t, *J* = 7.4 Hz, 12H), 3.57 (t, *J* = 8.1 Hz, 4H), 2.83 (hept, *J* = 6.9 Hz, 2H), 2.69 (t, *J* = 7.3 Hz, 4H), 2.04 (s, 6H), 1.81–1.76 (m, 4H), 1.67 (p, *J* = 7.6 Hz, 4H), 1.16 (d, *J* = 6.9 Hz, 12H). ^13^C NMR (126 MHz, DMSO-d6) δ 170.91, 148.89, 147.54, 133.71, 130.69, 128.97, 126.78, 123.81, 119.65, 65.56, 63.20, 50.49, 50.04, 32.76, 32.50, 23.72, 21.06, 20.77, 15.39.

4,4′-(1,4-Phenylenebis(methylene))bis(1-(11-(5-isopropyl-2-methylphenoxy)-11-oxoundecyl)-1,4-diazabicyclo[2.2.2]octane-1,4-diium) tetrabromide (**24**, DLpCAR-11). Prepared from compound **8** and *α*,*α*′-dibromo-*p*-xylene. Pale yellow solid, 76% yield. ^1^H NMR (400 MHz, DMSO-d6) δ 7.76 (s, 4H), 7.18 (dd, *J* = 7.8, 0.8 Hz, 2H), 7.04 (dd, *J* = 7.8, 1.8 Hz, 2H), 6.87 (d, *J* = 1.8 Hz, 2H), 5.00 (s, 4H), 4.01 (t, *J* = 7.6 Hz, 12H), 3.91 (t, *J* = 7.6 Hz, 12H), 3.47 (m, 4H), 2.83 (hept, *J* = 6.9 Hz, 2H), 2.58 (t, *J* = 7.4 Hz, 4H), 2.03 (s, 6H), 1.64 (p, *J* = 7.4 Hz, 8H), 1.40–1.20 (m, 24H), 1.16 (d, *J* = 6.9 Hz, 12H). ^13^C NMR (126 MHz, DMSO-d6) δ 171.39, 149.00, 147.53, 133.70, 130.66, 128.96, 126.79, 123.74, 119.63, 65.55, 63.61, 50.40, 50.03, 33.29, 32.75, 28.69, 28.63, 28.57, 28.40, 28.36, 25.45, 24.37, 23.71, 21.28, 15.29.

### 3.2. Determination of MICs

The bacterial strains (*Staphylococcus aureus* ATCC 25923, *Escherichia coli* ATCC 25922, *Salmonella enterica* ATCC 14028, *Citrobacter freundii* ATCC 8090, *Pseudomonas aeruginosa* ATCC 9027, *Acinetobacter baumannii* ATCC 19606) were obtained from the Collection of Extremophilic Microorganisms and Type Cultures (Knorre Institute of Chemical Biology and Fundamental Medicine SB RAS). Antimicrobial susceptibility testing was performed using the broth microdilution method in 96-well plates (TPP, Trasadingen, Switzerland), according to the guidelines of the European Committee on Antimicrobial Susceptibility Testing (EUCAST) [33]. A culture grown on Luria–Bertani (LB) agar (BD, Franklin Lakes, NJ, USA) was inoculated into Mueller–Hinton broth (MHB; OXOID, Basingstoke, UK) and incubated overnight. Stock solutions of the compounds in DMSO were prepared at a concentration of 20 mM. The final cell concentration of the overnight broth cultures was adjusted to approximately 5 × 10^5^ CFU/mL in MHB. Bacterial growth was assessed after 24 h by measuring the optical density at 595 nm for each well using a microplate reader (Uniplan, PICON, Moscow, Russia). The minimum inhibitory concentration (MIC)—defined as the lowest compound concentration that completely inhibited visible bacterial growth—was determined. All experiments were performed in triplicate, and the highest MIC value among the three replicates was selected for further analysis.

### 3.3. Time-Kill Kinetic Assay

A bacterial culture grown on LB agar was inoculated into MHB and incubated overnight. The resulting cell suspension was mixed with the tested compounds in MHB to achieve a final cell density of 1 × 10^5^ CFU/mL and then incubated at 37 °C. The compounds were evaluated at concentrations corresponding to their previously determined MICs. MHB without any compound served as the negative control. Bacterial aliquots (100 µL) were withdrawn at designated time intervals (0, 30, 60, 120, 240, and 360 min) and plated onto LB agar for colony counting [34].

### 3.4. RNA-Hydrolyzing Activity Assay

The RNA-hydrolyzing activity of the synthesized compounds was evaluated using a FAM-labeled 3′-end 17-mer ribooligonucleotide: 5′-AGUUACCUCGGAUGCAA-3′-FAM, synthesized and kindly provided by Dr. M.I. Meschaninova (Knorre Institute of Chemical Biology and Fundamental Medicine SB RAS).

RNA target was added to antimicrobials **9**–**24** at final concentrations of 1 and 10 μM in a buffer solution containing 50 mM Tris-Acetate, pH 7.4, and 100 mM NaCl. The total volume of probes was 50 μL. The mixture was incubated at 37 °C for 6 h, followed by sample precipitation. After the addition of 1× denaturing dye for electrophoresis (5 μL; 8 M urea, XC), 2.5 μL of the reaction mixture containing the products of RNA hydrolysis was analyzed by denaturing electrophoresis in 20% PAAG. Gels were scanned using a Gel Doc XR+ System (Bio-Rad, Hercules, CA, USA) at the excitation wavelength of 488 nm and an emission filter of 530 nm; the signal intensity was estimated using the Image Lab™ 6.0.1 software package (Bio-Rad, USA). The extent of the cleavage was calculated as the ratio of the intensity of the defined band to the total intensity of all bands in the lane. The relative error in all experiments did not exceed 20%.

### 3.5. Hemolytic Activity Assay

The hemolytic activity of the synthesized compounds was evaluated using rabbit red blood cells (RBCs) according to published techniques [35,36,37]. Fresh rabbit venous blood collected with heparin was centrifuged at 520 *g* for 5 min, the plasma was removed, and the RBCs were washed three times with 0.9% NaCl cooled to 4 °C. RBCs were diluted to 8% *v*/*v* in 0.9% NaCl and added to aliquots containing varying concentrations of the test compounds in a 1:1 ratio, resulting in a final RBC concentration of 4% *v*/*v* in all samples. The samples were incubated for 30 min at 37 °C and centrifuged at 2000 *g* for 5 min. The supernatant was collected, and the amount of released hemoglobin was determined by measuring absorbance at 414.5 nm (UV-1800 spectrometer, Shimadzu, Kyoto, Japan). RBCs suspended in 0.9% NaCl alone served as the negative control (0% hemolysis), while RBCs treated with 5% Triton X-100 (Merck, Germany) aqueous solution served as the positive control (100% hemolysis). All experiments were performed in triplicate. The percentage of hemolysis was calculated using the following equation:

$$Hemolysis\;\left(\%\right)=\;\frac{{OD}_{414.5}\;sample-\;{OD}_{414.5}\;0\%\;lysis}{{OD}_{414.5}\;100\%\;lysis-{OD}_{414.5}\;0\%\;lysis}\times 100\%$$

### 3.6. MTT Cell Viability Assay

RPMI8226 human myeloma cell lines and LMTK mouse fibroblasts were obtained from the Collection of Cell Cultures (Knorre Institute of Chemical Biology and Fundamental Medicine SB RAS). MEF primary cells (mouse embryonic fibroblasts) were isolated from mouse embryos with a generic background of C57BL/6J mice (kindly provided by E.N. Kozhevnikova, Institute of Cytology and Genetics SB RAS) according to the protocol [38]. MEF and LMTK cells were incubated in Dulbecco’s modified Eagle’s medium (DMEM; Gibco, Grand Island, NY, USA) supplemented with 10% fetal bovine serum (FBS; Gibco, USA), 100 IU/mL of penicillin and 100 μg/mL of streptomycin (Hyclone, Thermo Scientific, Logan, UT, USA) and 0.37% NaHCO_3_ at 37 °C in a humidified atmosphere of 95% air and 5% CO_2_. The cells were trypsinized in 0.05% Trypsin-EDTA (Gibco, USA). RPMI8226 cells were incubated in RPMI Medium 1640 (Gibco, USA) supplemented with 10% fetal bovine serum (FBS; Gibco, USA), 100 IU/mL of penicillin and 100 μg/mL of streptomycin (Hyclone, Thermo Scientific, USA) and 0.37% NaHCO_3_ at 37 °C in a humidified atmosphere of 95% air and 5% CO_2_. RPMI8226 and LMTK cells were seeded at 20,000 cells per well in 96-well plates. MEF cells were seeded at 10,000 cells per well at passage 2. After 24 h of incubation, the culture medium was removed and the tested compound was added to the culture medium at 4–500 µM doses. Camptothecin (MP Biomedicals, Irvine, CA, USA) was used as a positive control. The stock solutions of the compounds were prepared in PBS and further dilutions were made with fresh medium. The stock solution of camptothecin was prepared in DMSO, and its final concentration was below 0.1%. All experiments were performed in technical triplicates across three independent biological replicates. Cell viability was calculated as a percentage relative to the untreated control group. After 72 h of incubation, the cytotoxicity test was performed using the MTT assay. A 20 μL volume of MTT (MP Biomedicals, USA) solution in PBS (5 mg/mL) was added to each well and plates were left in a cell incubator for 4 h at 37 °C, 5% CO_2_ to allow cells to metabolize yellow MTT to blue formazan. Then lysing mixture, consisting of 5.5 g of sodium dodecyl sulphate, 25 mL of dimethylformamide, and 30 mL of distilled water, was added at a volume of 100 μL per well. Plates were incubated for 24 h to dissolve formazan crystals. OD of the plate was read at 570 nm with a reference wavelength at 620 nm. Inhibition was calculated for each concentration of the compounds. Data and estimated IC_50_ values are expressed as the mean ± SD of three independent experiments. Statistical analyzes and non-linear regressions were performed using GraphPad Prism version 8.0.

## 4. Conclusions

In this study, novel amphiphilic carvacrol–DABCO hybrids were designed and synthesized. It was demonstrated that the introduction of carvacrol instead of alkyl tails significantly enhances selectivity compared to previously reported compounds, while the polycationic amphiphilic scaffold preserves a dual-target mode of action by driving bacterial membrane disruption and RNA cleavage.

Despite these findings, certain limitations of the current study warrant consideration. The biological screening was confined to standard reference strains in their planktonic state, meaning that future research must prioritize extensive in vitro evaluations against multidrug-resistant (MDR) clinical isolates and mature bacterial biofilms, accompanied by serial passage assays to determine the frequency and probability of bacterial resistance development to these agents. Additionally, given that these structures function as cationic surfactants, their physicochemical behavior remains uncharacterized, necessitating the precise determination of the critical micelle concentration to establish how self-assembly properties dictate their biological activities. Investigating the structural stability of the ester linkage against plasma esterases, defining the sequence-specificity of the RNA-cleavage mechanism, and conducting in vivo acute toxicity studies represent key steps to further assess the translational potential of these carvacrol–DABCO hybrids.

## Figures and Tables

**Table 1:** MIC values and structural parameters of the synthesized compounds.

Compound	MIC, μM						Central Linker Length, Å *	Lateral Linker Length, Å *
	*S. aureus*	*E. coli*	*S. enterica*	*C. freundii*	*P. aeruginosa*	*A. baumannii*		
Carvacrol	2000	2000	2000	2000	>8000	1000		
Ciprofloxacin	2	0.31	0.31	0.31	0.5	2		
DL4-12	4	8	16	4	125	8	6.4	
(**6**) DCAR-5	4000	8000	8000	4000	8000	8000		
(**9**) DL4CAR-4	8	31	125	16	63	1000	6.4	6.4
(**10**) DL4CAR-5	16	31	63	63	250	500		7.4
(**11**) DL4CAR-6	4	8	16	8	16	250		8.7
(**12**) DL4CAR-10	31	63	250	63	500	500		14.9
(**13**) DL5CAR-4	31	63	250	63	125	500	7.4	6.4
(**14**) DL5CAR-5	16	16	63	16	125	250		7.4
(**15**) DL5CAR-6	8	16	63	31	16	250		8.7
(**16**) DL5CAR-10	16	31	125	125	125	250		14.9
(**17**) DLoCAR-4	16	31	125	31	500	1000	5.4	6.4
(**18**) DLoCAR-5	8	31	125	63	500	1000		7.4
(**19**) DLoCAR-6	8	31	125	31	250	500		8.7
(**20**) DLoCAR-10	16	31	63	250	500	125		14.9
(**21**) DLpCAR-4	8	63	250	63	63	>1000	7.8	6.4
(**22**) DLpCAR-5	8	31	63	63	16	1000		7.4
(**23**) DLpCAR-6	8	16	63	31	31	500		8.7
(**24**) DLpCAR-10	8	31	125	31	63	125		14.9

* Calculated by using Chem 3D Ultra 16.0v.

**Table 2:** Hemolytic activity of the synthesized compounds and DL4-12.

Compound	Concentration, µM				
	32	63	125	250	500
	Hemolysis, %				
DL4-12	74.2 ± 2.6	ND	ND	ND	ND
(**9**) DL4CAR-4	0.31 ± 0.09	1.09 ± 0.11	2.35 ± 0.13	4.6 ± 0.5	10.4 ± 0.4
(**10**) DL4CAR-5	0.33 ± 0.13	0.75 ± 0.07	1.74 ± 0.09	3.14 ± 0.18	6.4 ± 0.5
(**11**) DL4CAR-6	1.37 ± 0.23	2.88 ± 0.19	6.2 ± 0.3	17.6 ± 2.5	41.6 ± 1.5
(**13**) DL5CAR-4	0.12 ± 0.07	0.63 ± 0.06	2.14 ± 0.09	2.87 ± 0.17	4.26 ± 0.23
(**14**) DL5CAR-5	0.41 ± 0.10	0.66 ± 0.06	1.95 ± 0.12	3.87 ± 0.16	4.28 ± 0.19
(**15**) DL5CAR-6	1.28 ± 0.07	1.75 ± 0.12	2.1 ± 0.4	4.6 ± 0.3	10.0 ± 0.5
(**17**) DLoCAR-4	<0.1	0.97 ± 0.09	1.5 ± 0.1	2.67 ± 0.08	4.2 ± 0.3
(**18**) DLoCAR-5	<0.1	1.67 ± 0.06	2.89 ± 0.24	5.3 ± 1.1	6.1 ± 0.3
(**19**) DLoCAR-6	<0.1	4.06 ± 0.11	6.40 ± 0.21	8.1 ± 0.3	17.5 ± 2.1
(**21**) DLpCAR-4	<0.1	0.93 ± 0.20	0.78 ± 0.15	1.41 ± 0.15	2.25 ± 0.21
(**22**) DLpCAR-5	<0.1	0.95 ± 0.09	1.2 ± 0.3	0.84 ± 0.11	1.5 ± 0.3
(**23**) DLpCAR-6	<0.1	1.26 ± 0.13	1.14 ± 0.04	1.19 ± 0.20	3.45 ± 0.21

Data are presented as mean ± SD.

**Table 3:** IC_50_ values of the compounds DL4-12, DL4CAR-6 and DL5CAR-6 against different cell lines.

Compound	IC_50_, µM		
	RPMI8226	LMTK	MEF
DL4-12	22 ± 5	37 ± 5	24 ± 5
DL4CAR-6	250 ± 20	202 ± 22	114 ± 11
DL5CAR-6	213 ± 15	185 ± 17	117 ± 11
Campotothecin	5.9 ± 1.0	34 ± 6	17 ± 4

Data are presented as mean ± SD.

## Data Availability

Data are available in the article and the Supplementary Materials.

## Supplementary Materials

The following supporting information can be downloaded at: https://www.mdpi.com/article/10.3390/antibiotics15090927/s1, Figure S1. ^1^H and ^13^C NMR spectra of CAR-4 (**1**); Figure S2. ^1^H and ^13^C NMR spectra of CAR-5 (**2**); Figure S3. ^1^H and ^13^C NMR spectra of CAR-11 (**4**); Figure S4. ^1^H and ^13^C NMR spectra of DCAR-4 (**5**); Figure S5. ^1^H and ^13^C NMR spectra of DCAR-5 (**6**); Figure S6. ^1^H and ^13^C NMR spectra of DCAR-11 (**8**); Figure S7. ^1^H and ^13^C NMR spectra of DL4CAR-4 (**9**); Figure S8. ^1^H and ^13^C NMR spectra of DL4CAR-5 (**10**); Figure S9. ^1^H and ^13^C NMR spectra of DL4CAR-11 (**12**); Figure S10. ^1^H and ^13^C NMR spectra of DL5CAR-4 (**13**); Figure S11. ^1^H and ^13^C NMR spectra of DL5CAR-5 (**14**); Figure S12. ^1^H and ^13^C NMR spectra of DL5CAR-11 (**16**); Figure S13. ^1^H and ^13^C NMR spectra of DLoCAR-4 (**17**); Figure S14. ^1^H and ^13^C NMR spectra of DLoCAR-5 (**18**); Figure S15. ^1^H and ^13^C NMR spectra of DLoCAR-11 (**20**); Figure S16. ^1^H and ^13^C NMR spectra of DLpCAR-4 (**21**); Figure S17. ^1^H and ^13^C NMR spectra of DLpCAR-5 (**22**); Figure S18. ^1^H and ^13^C NMR spectra of DLpCAR-11 (**24**); Figure S19. Mass spectrum of DCAR-4 (**5**); Figure S20. Mass spectrum of DCAR-5 (**6**); Figure S21. Mass spectrum of DCAR-11 (**8**).

## Abbreviations

The following abbreviations are used in this manuscript:

DABCO	1,4-Diazabicyclo[2.2.2]octane
MIC	Minimum inhibitory concentration
IC_50_	Half-maximal inhibitory concentration
TLC	Thin-layer chromatography
DCM	Dichloromethane
LB	Luria–Bertani
MHB	Mueller–Hinton broth
RBC	Red blood cell
