# Supplementary material for: Synthesis and In Vitro Biological Evaluation of Amphiphilic Derivatives of Carvacrol as Potent Antibacterial Agents
Source: Antibiotics (Basel). 2026 Sep 18;15(9):927. doi: 10.3390/antibiotics15090927 (PMC13603598; doi:10.3390/antibiotics15090927)
Supplement: Supplementary file 1 [file antibiotics-15-00927-s001.zip › antibiotics-4493928-supplementary.pdf]

1. ^{1}H and ^{13}C NMR spectra

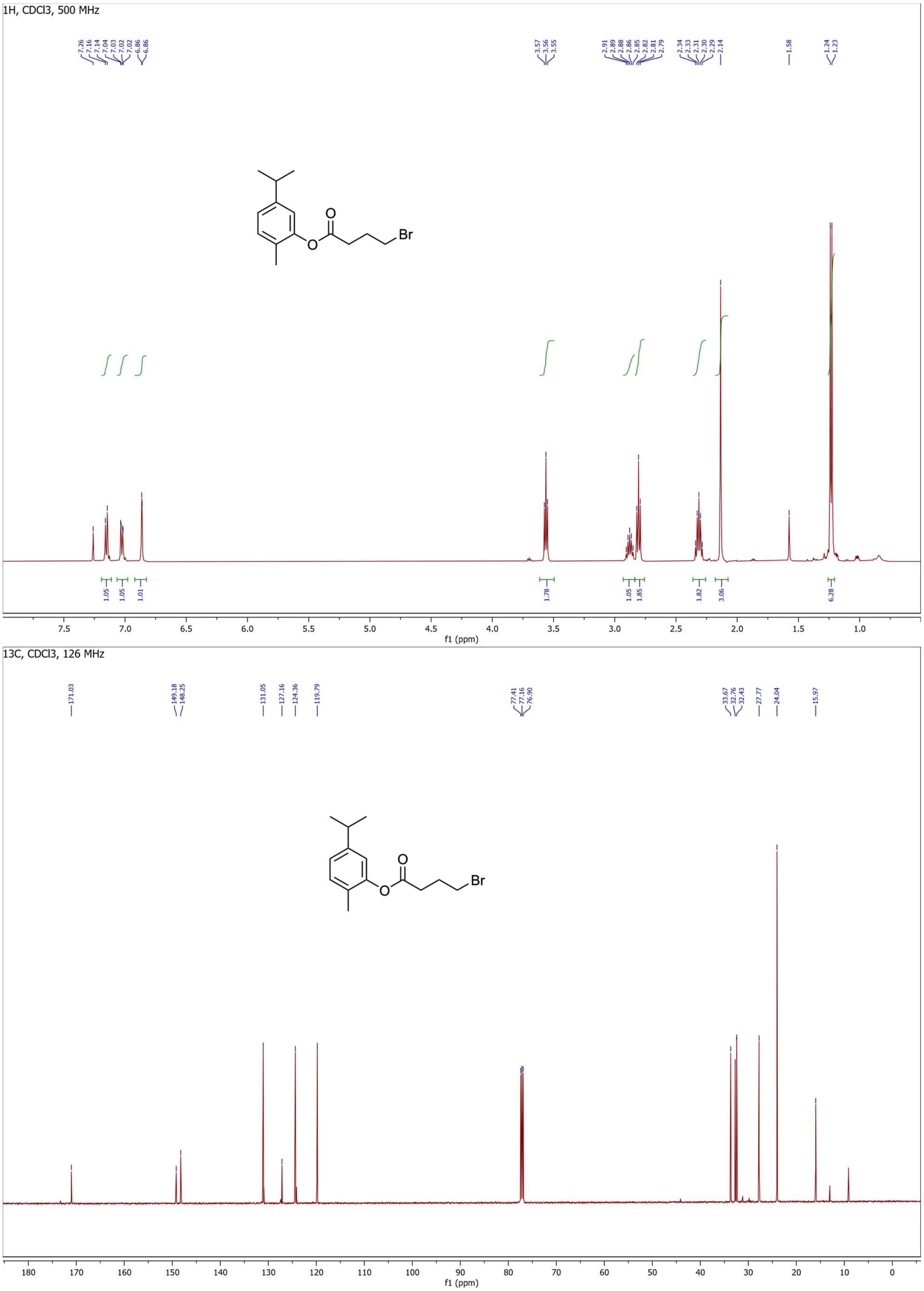

Figure S1. ^{1}H and ^{13}C NMR spectra of CAR-4 (**1**).

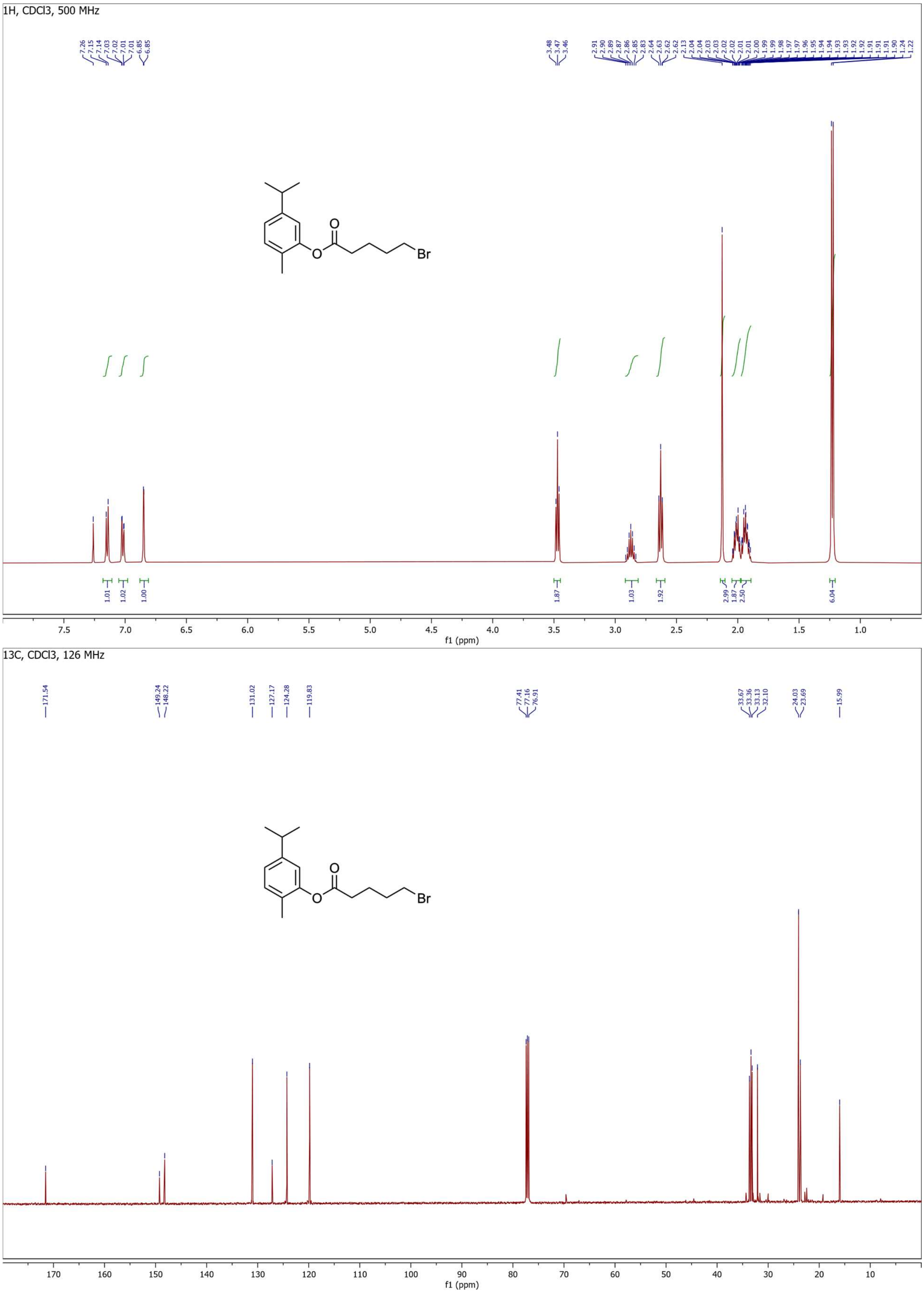

Figure S2. ^{1}H and ^{13}C NMR spectra of CAR-5 (**2**).

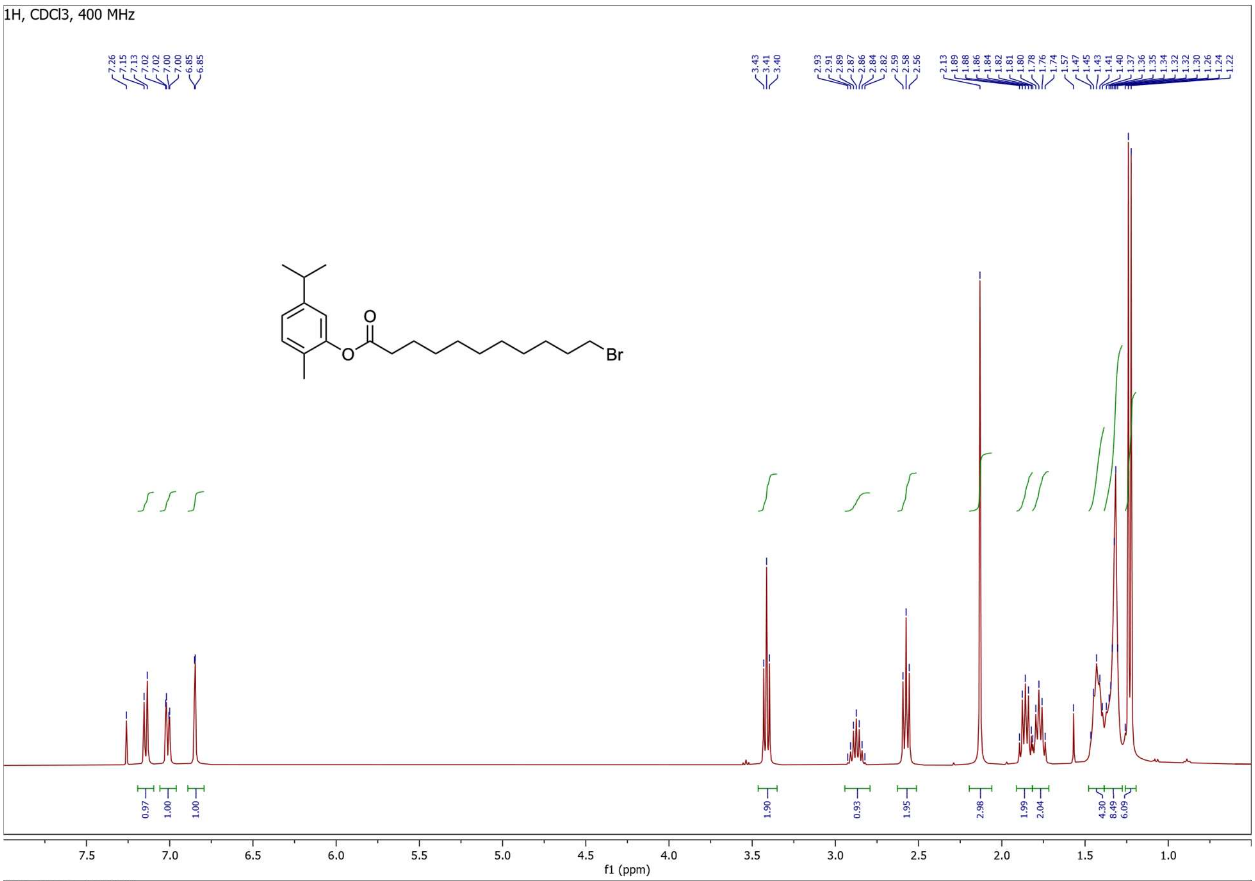

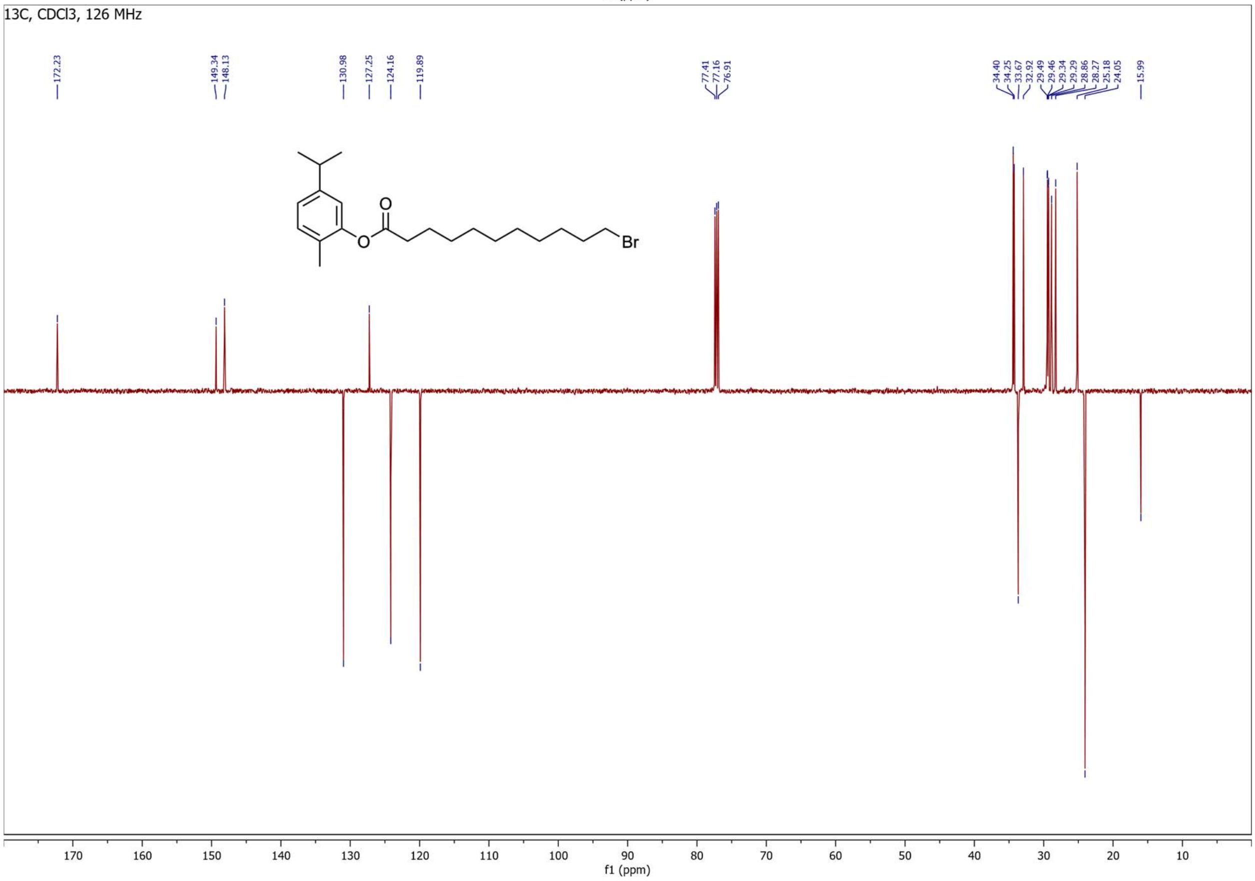

Figure S3. ^{1}H and ^{13}C NMR spectra of CAR-11 (**4**).

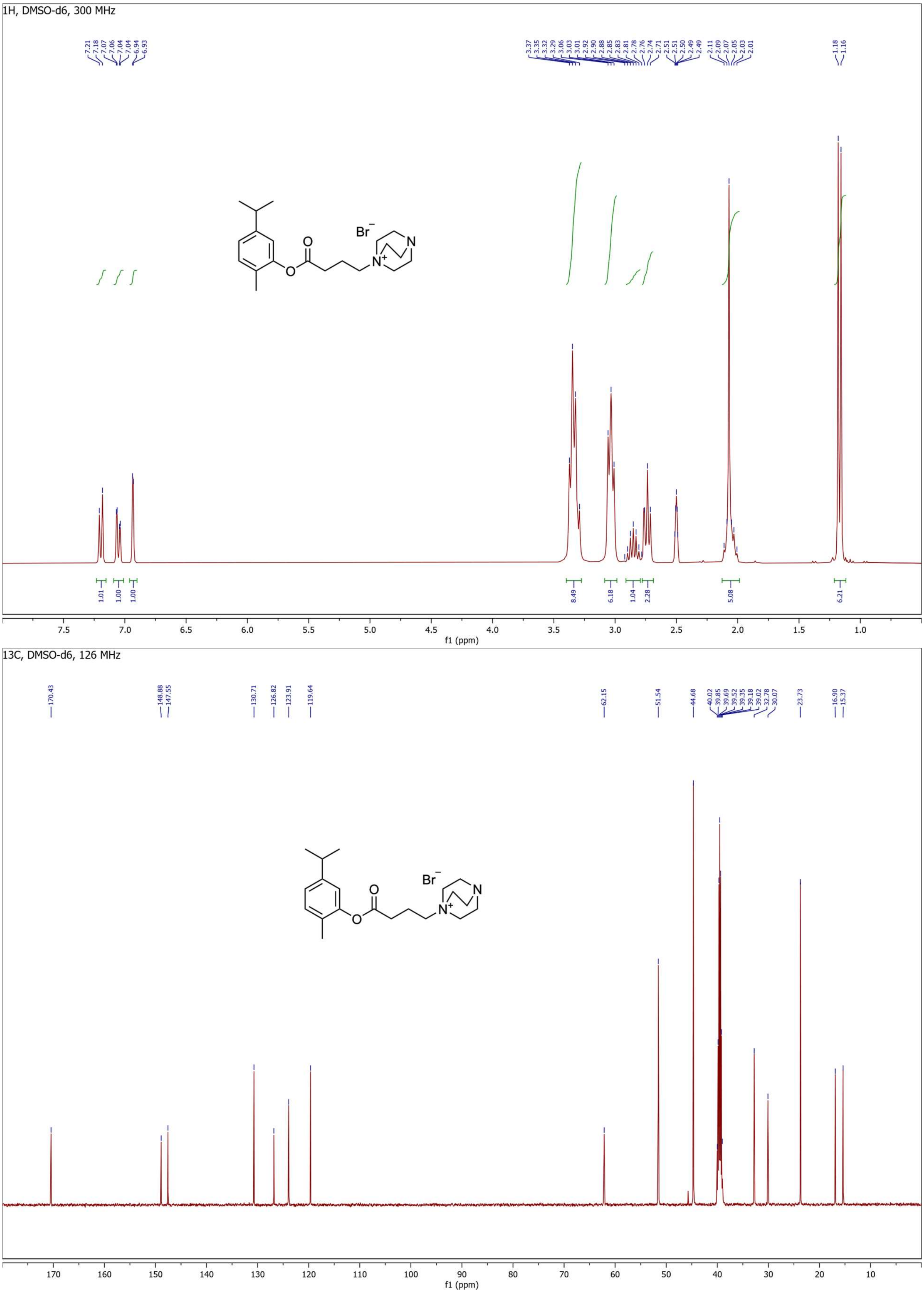

Figure S4. ^{1}H and ^{13}C NMR spectra of DCAR-4 (**5**).

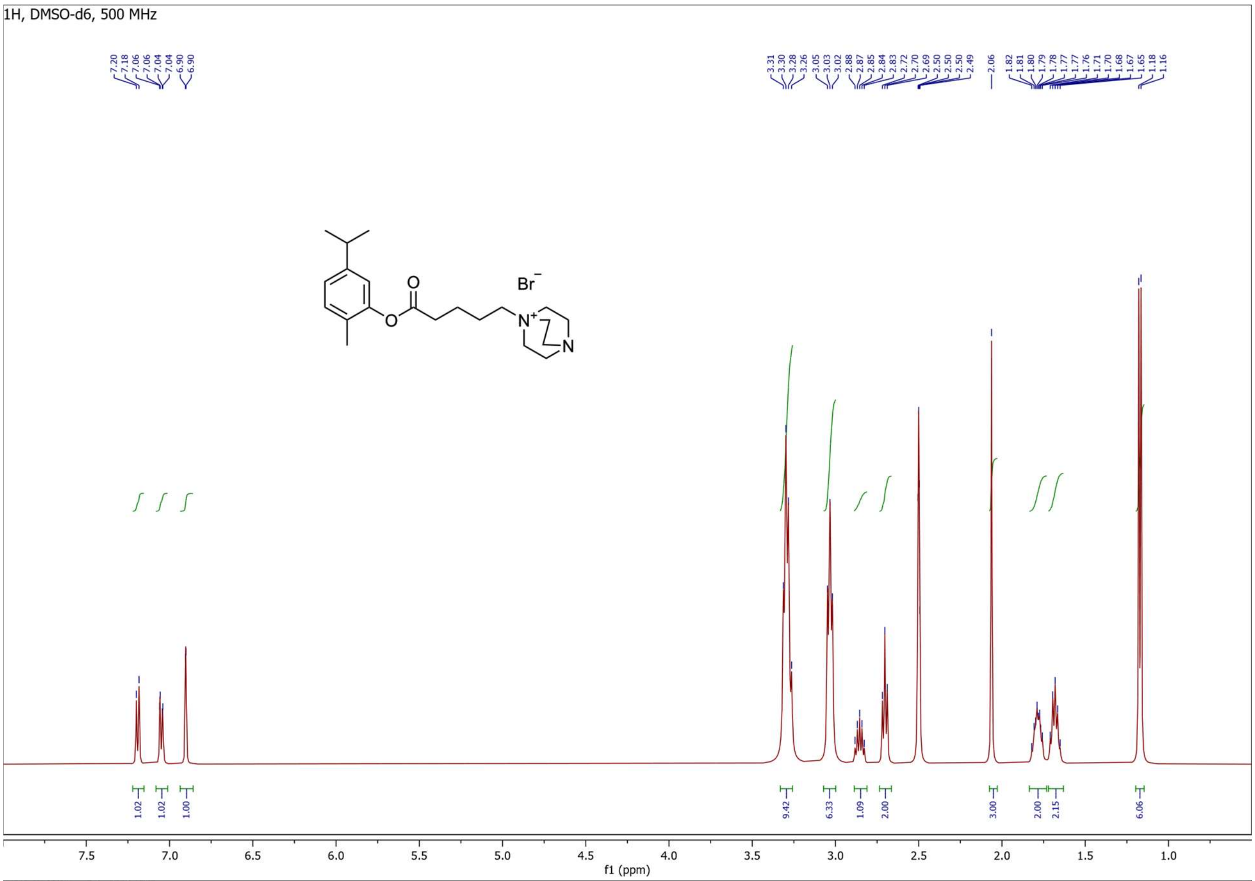

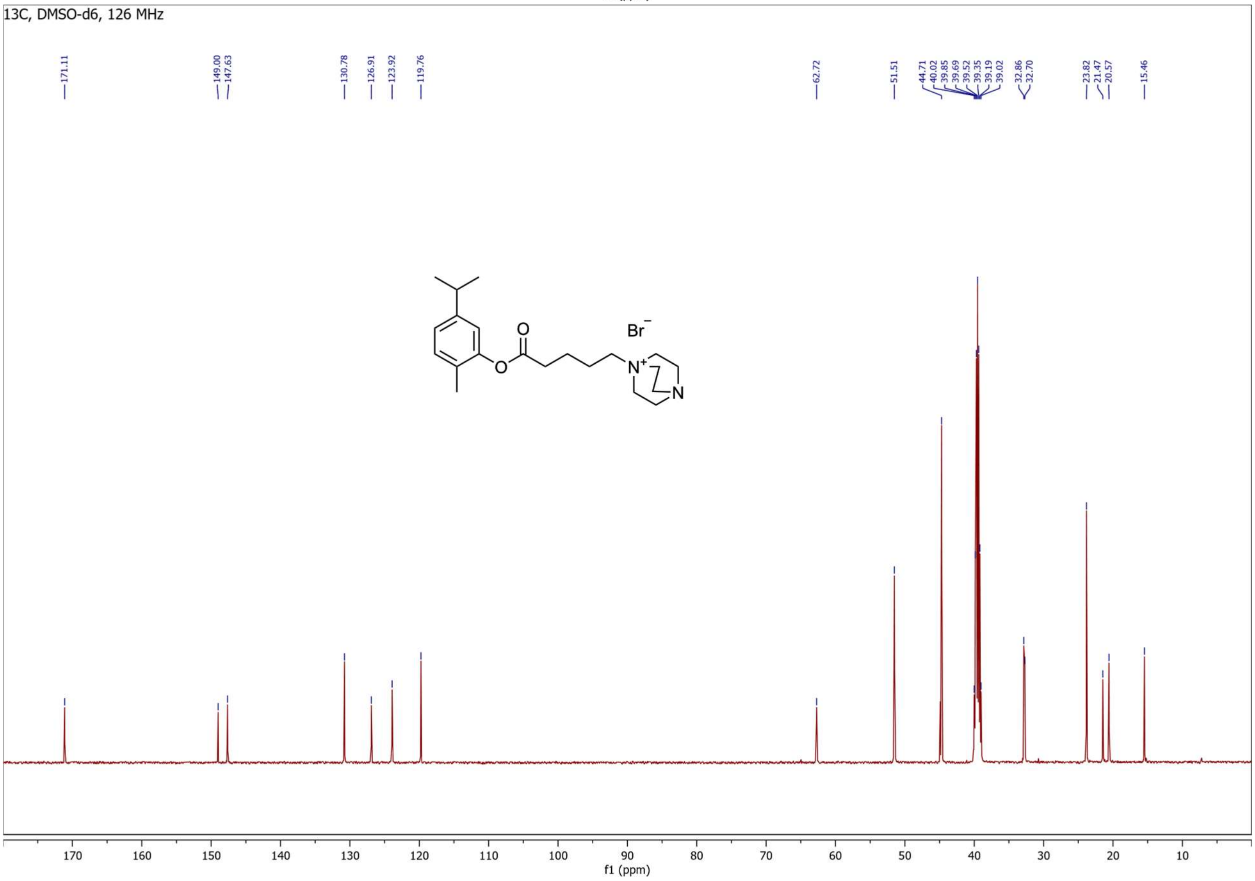

Figure S5. ^{1}H and ^{13}C NMR spectra of DCAR-5 (**6**).

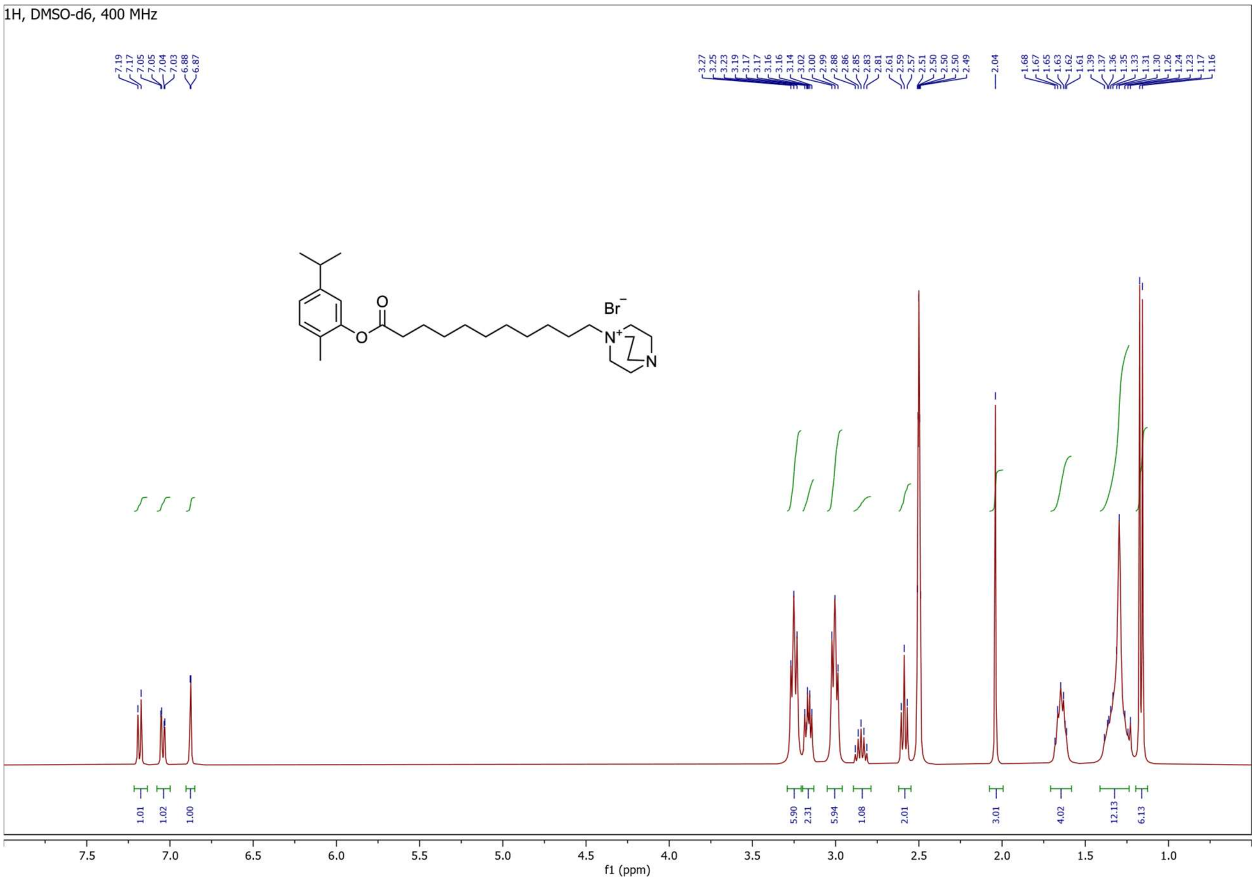

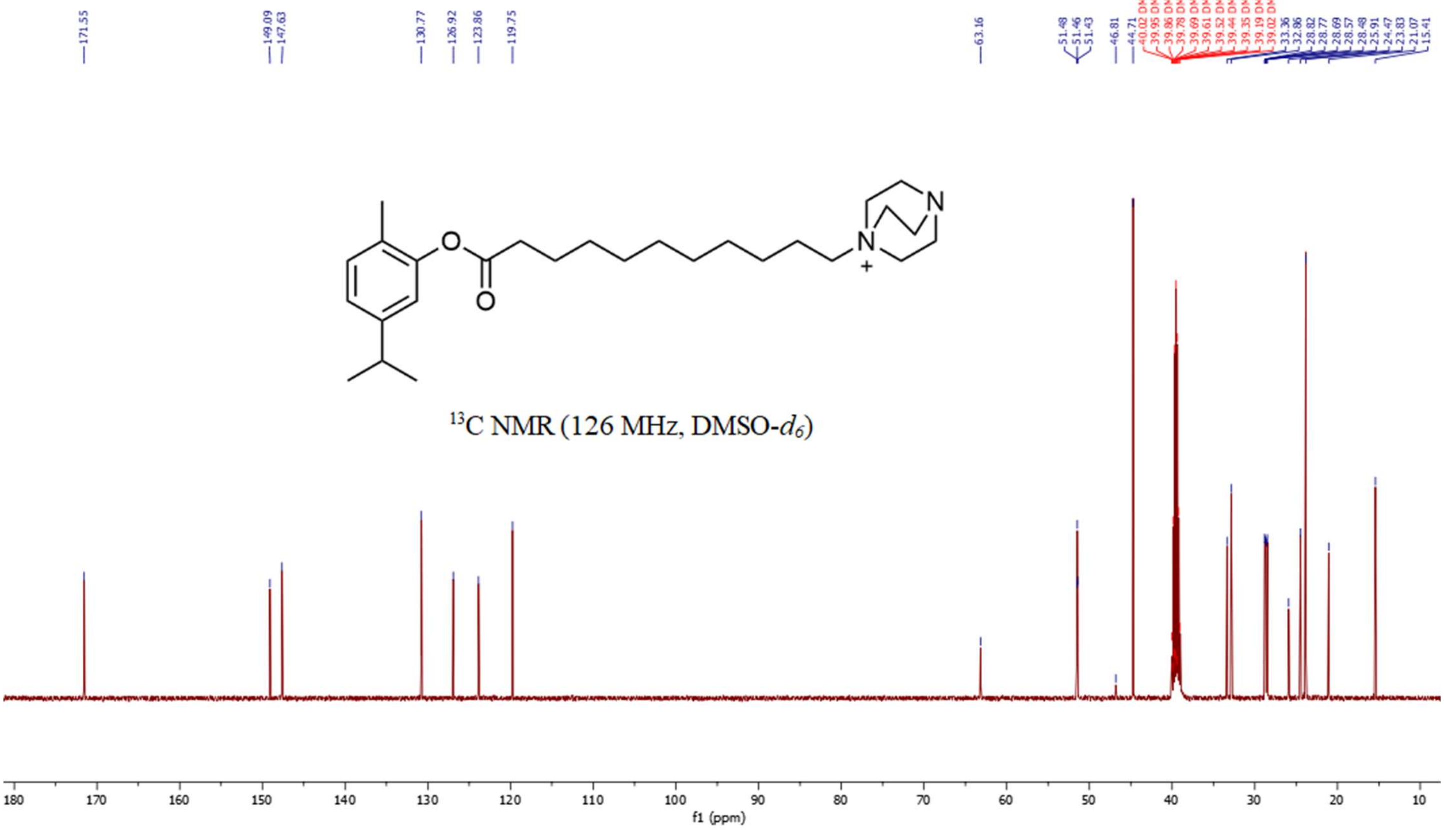

Figure S6. ^{1}H and ^{13}C NMR spectra of DCAR-11 (**8**).

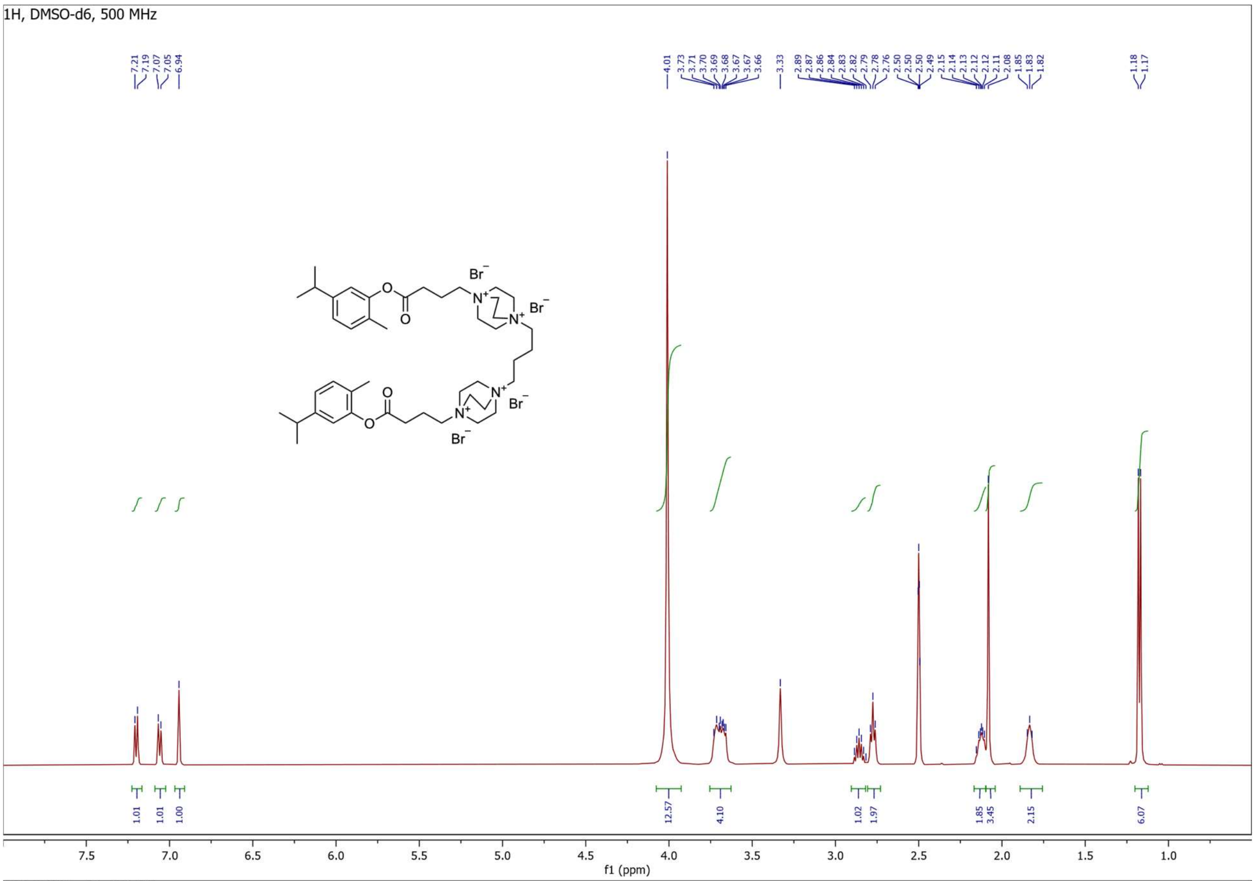

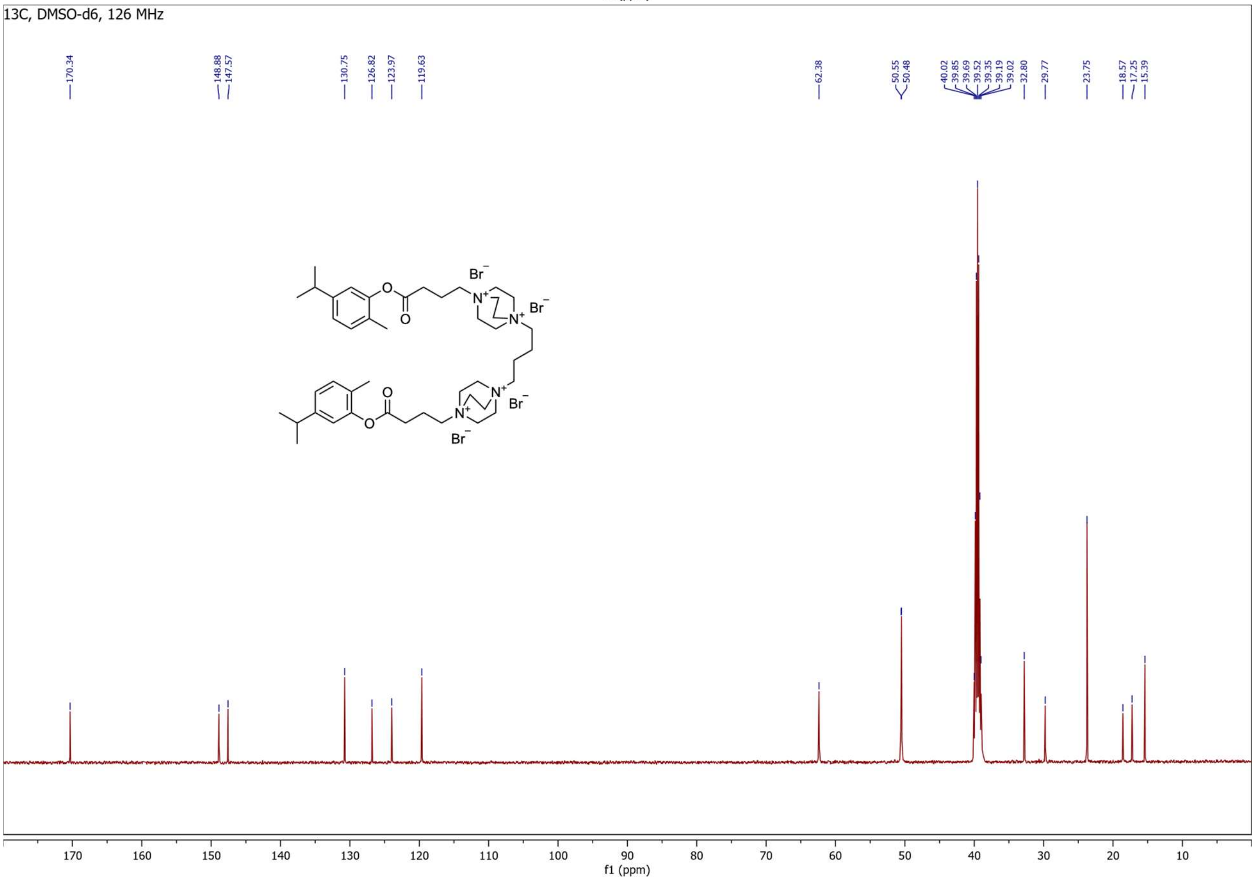

Figure S7. ^{1}H and ^{13}C NMR spectra of DL4CAR-4 (**9**).

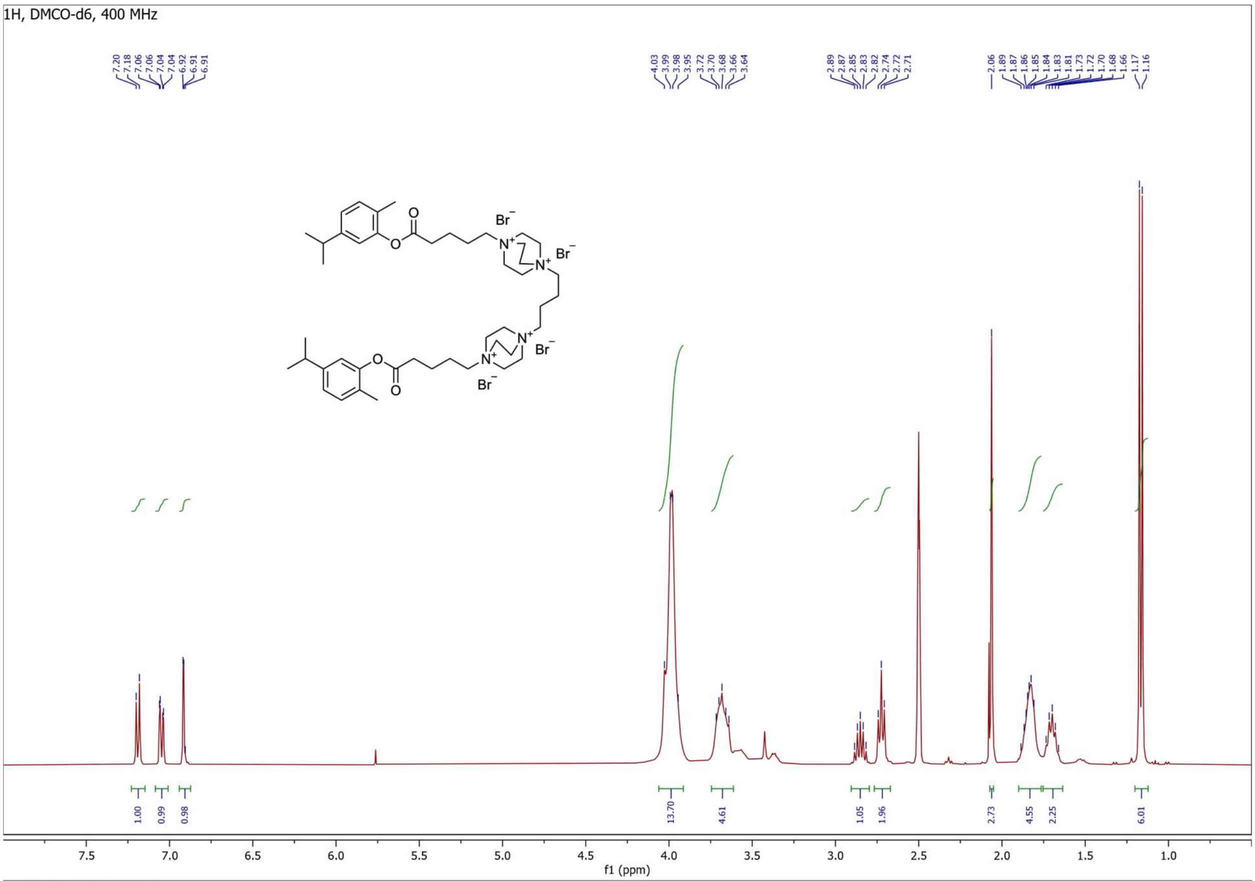

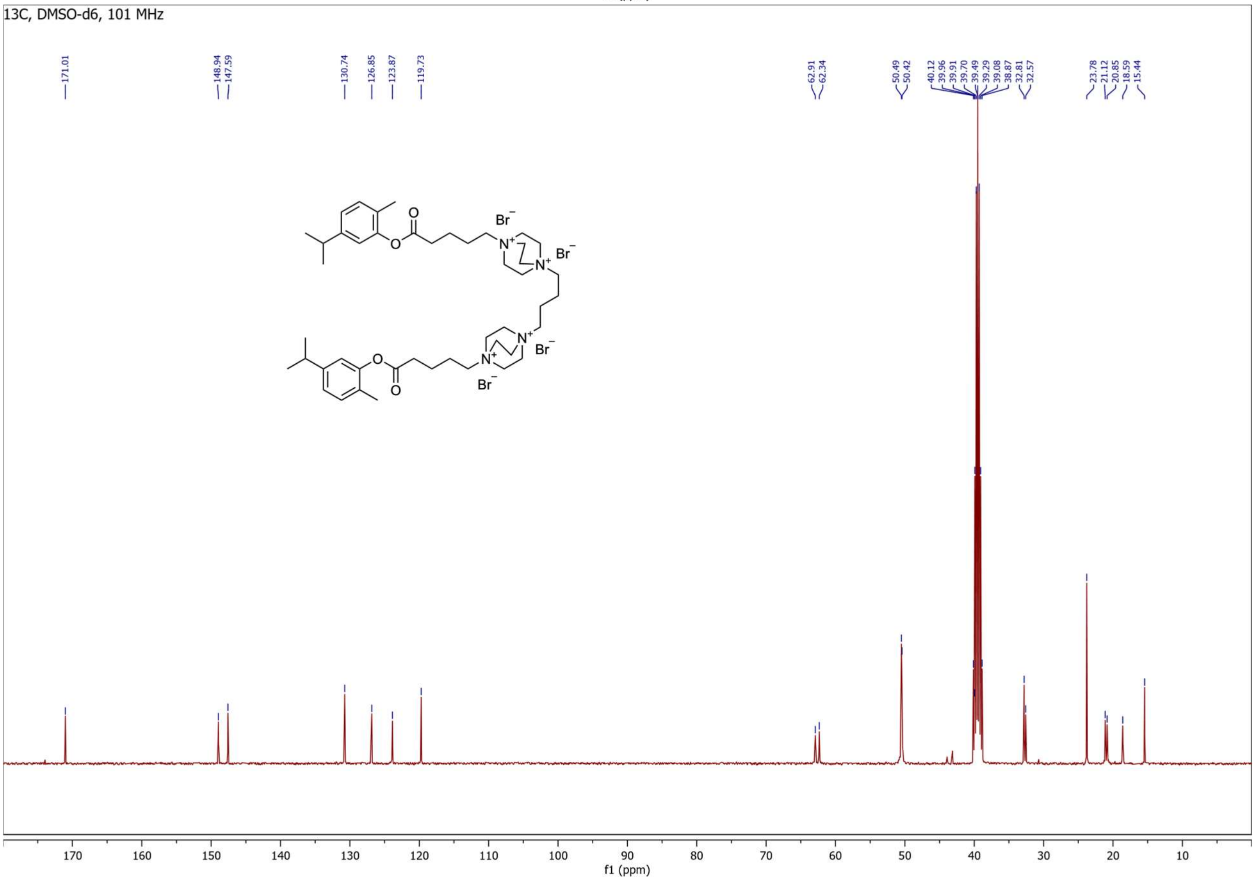

Figure S8. ^{1}H and ^{13}C NMR spectra of DL4CAR-5 (**10**).

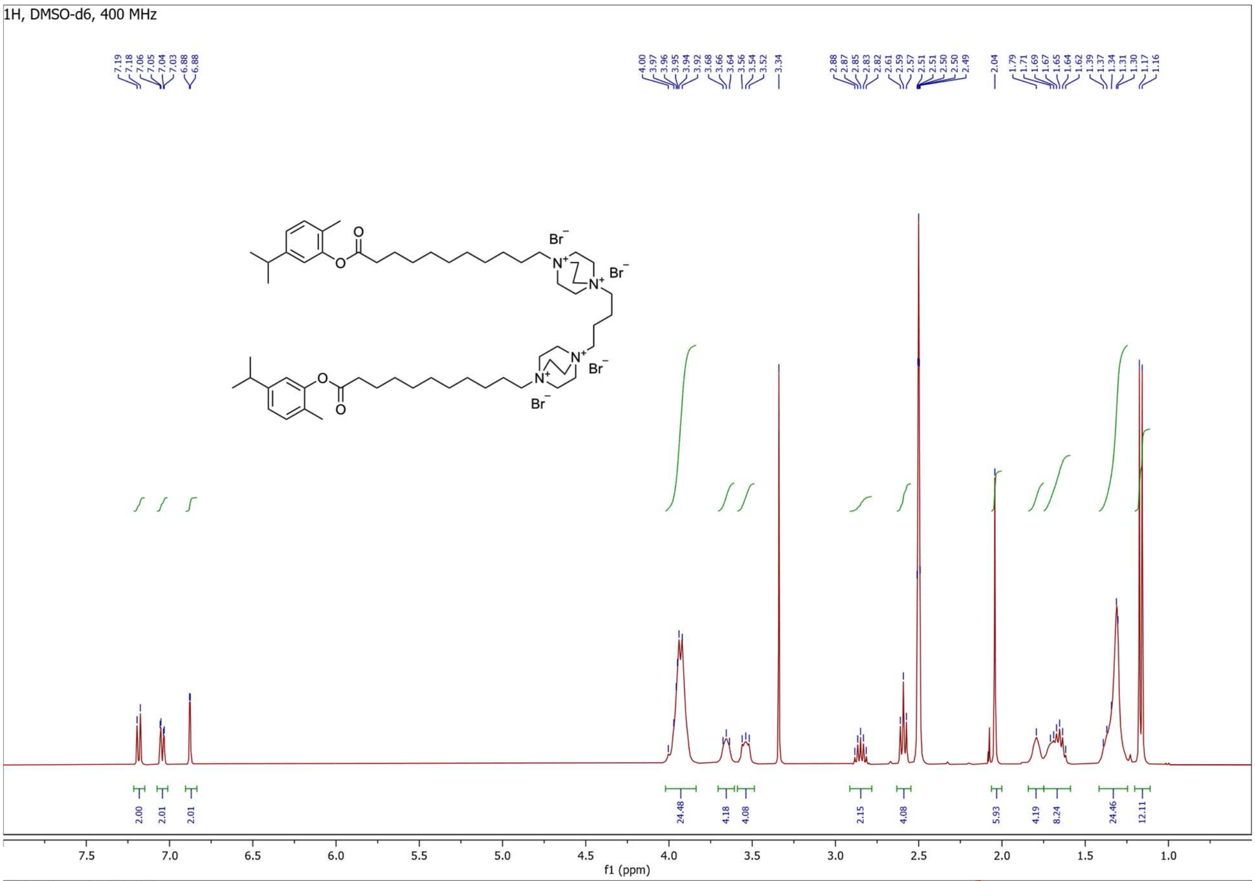

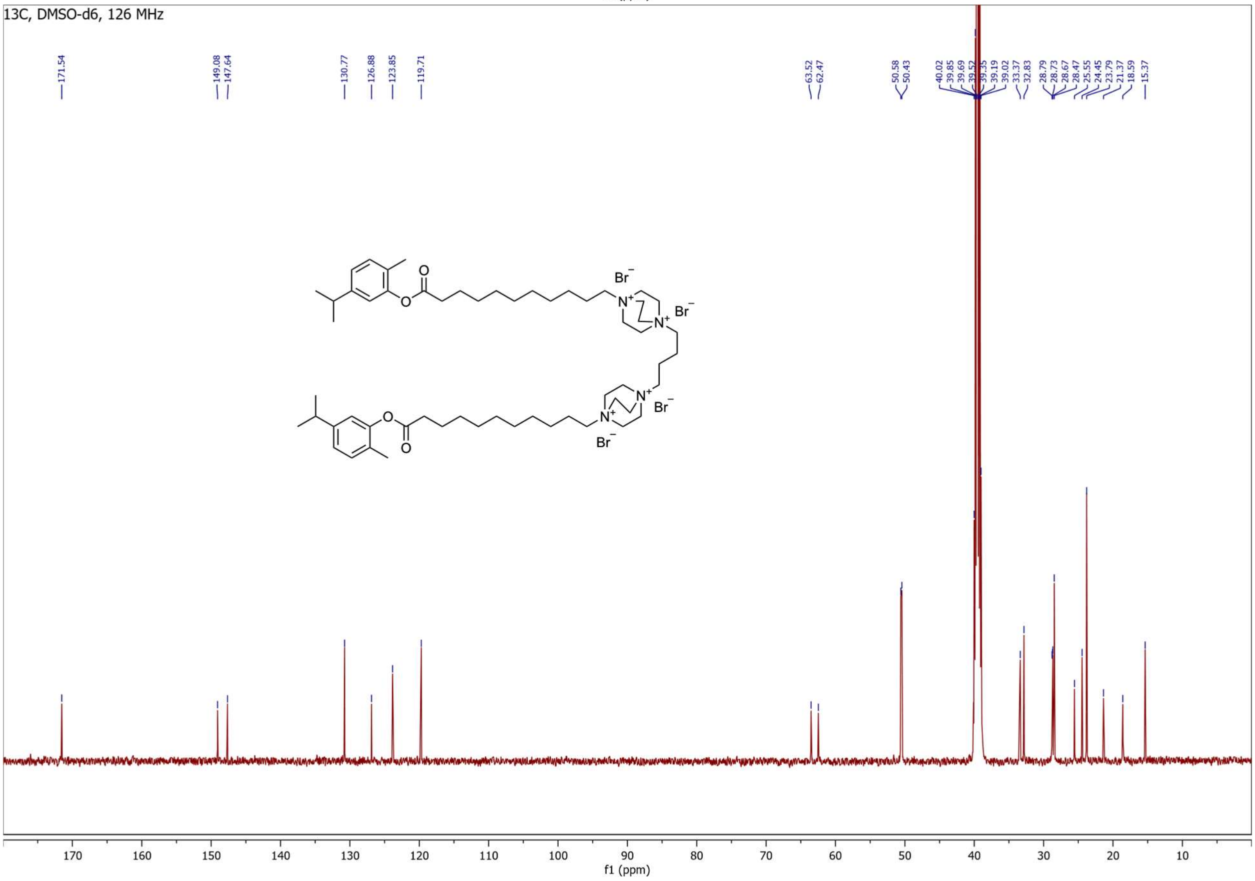

Figure S9. ^{1}H and ^{13}C NMR spectra of DL4CAR-11 (**12**).

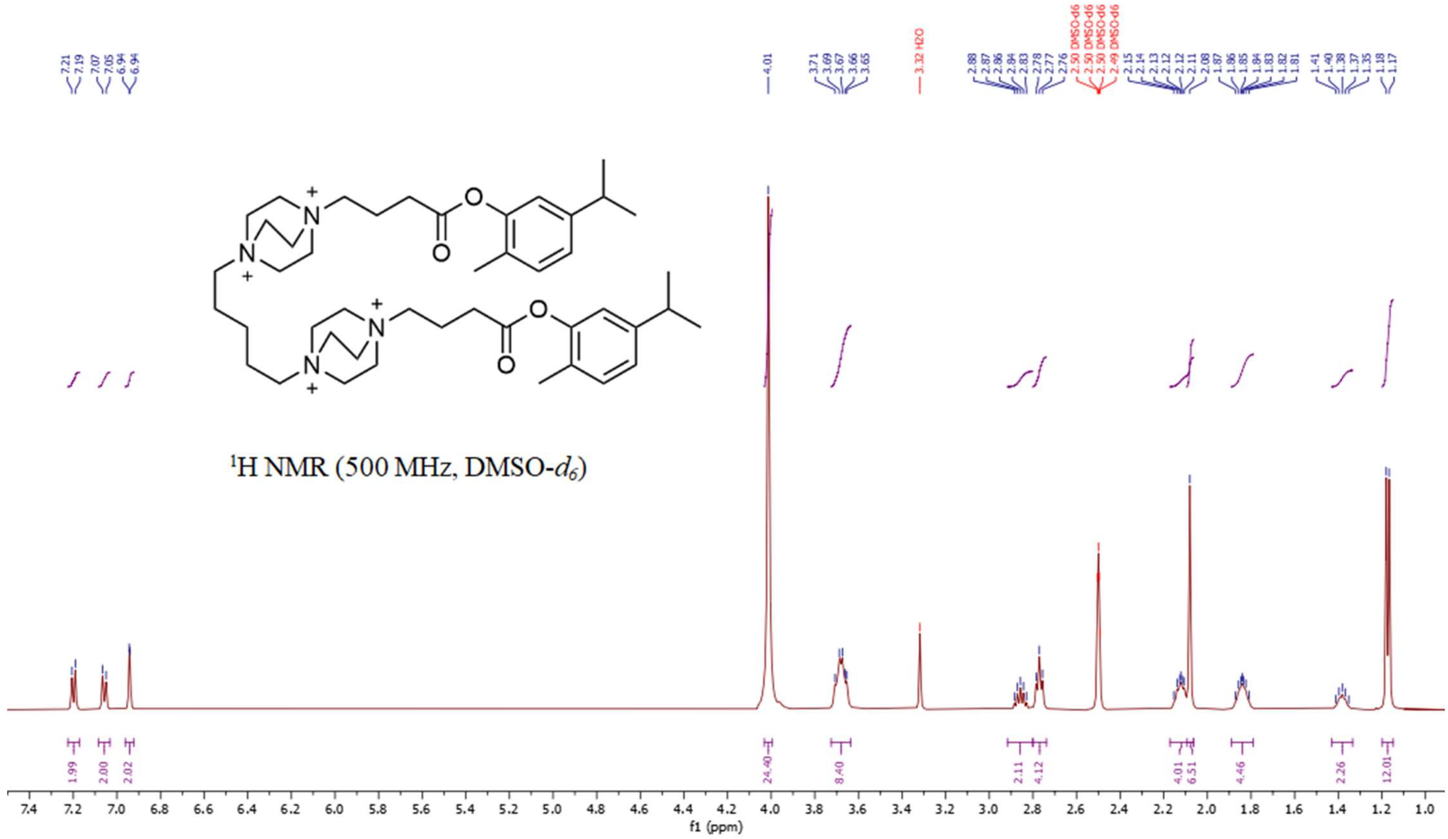

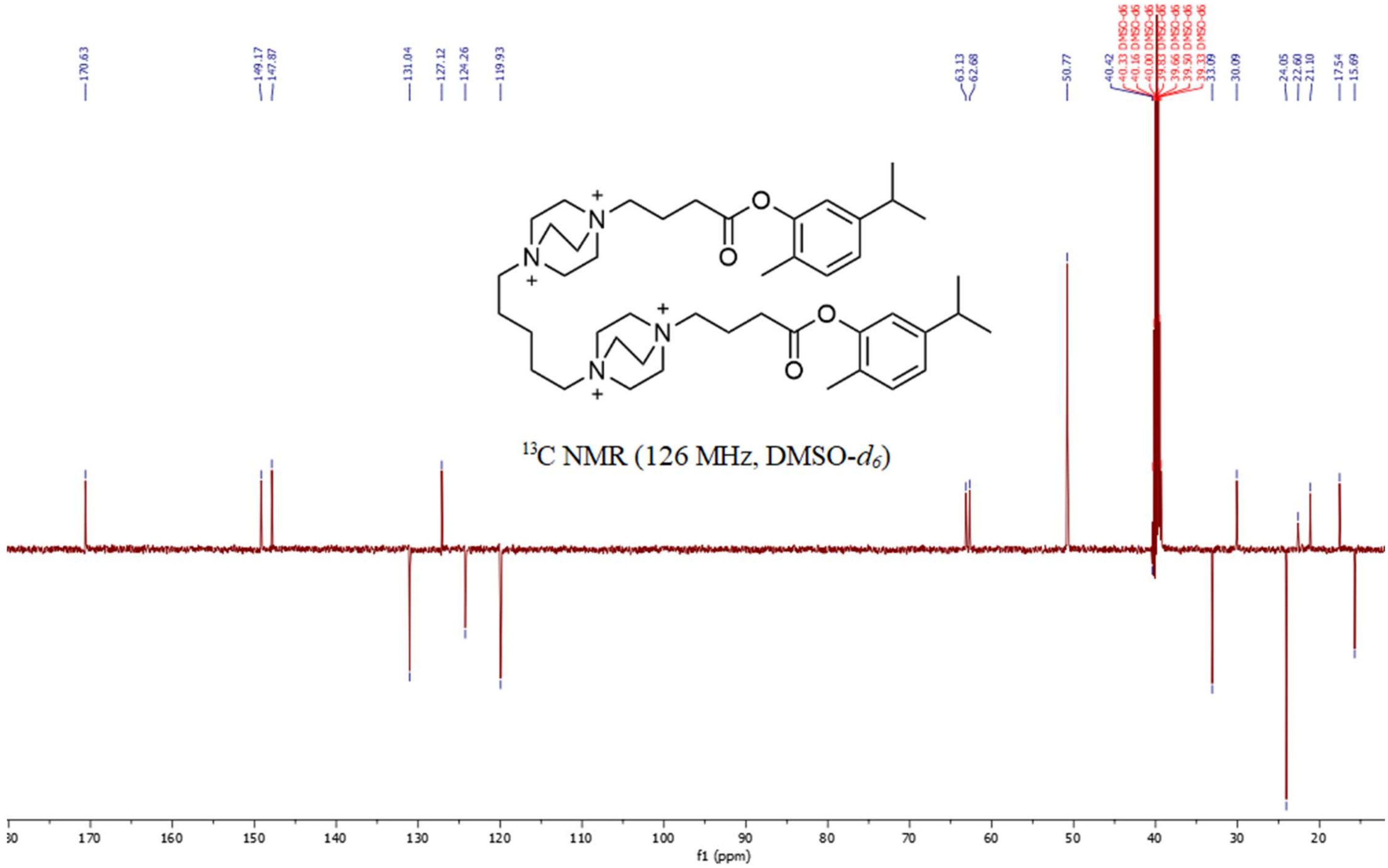

Figure S10. ^{1}H and ^{13}C NMR spectra of DL5CAR-4 (**13**).

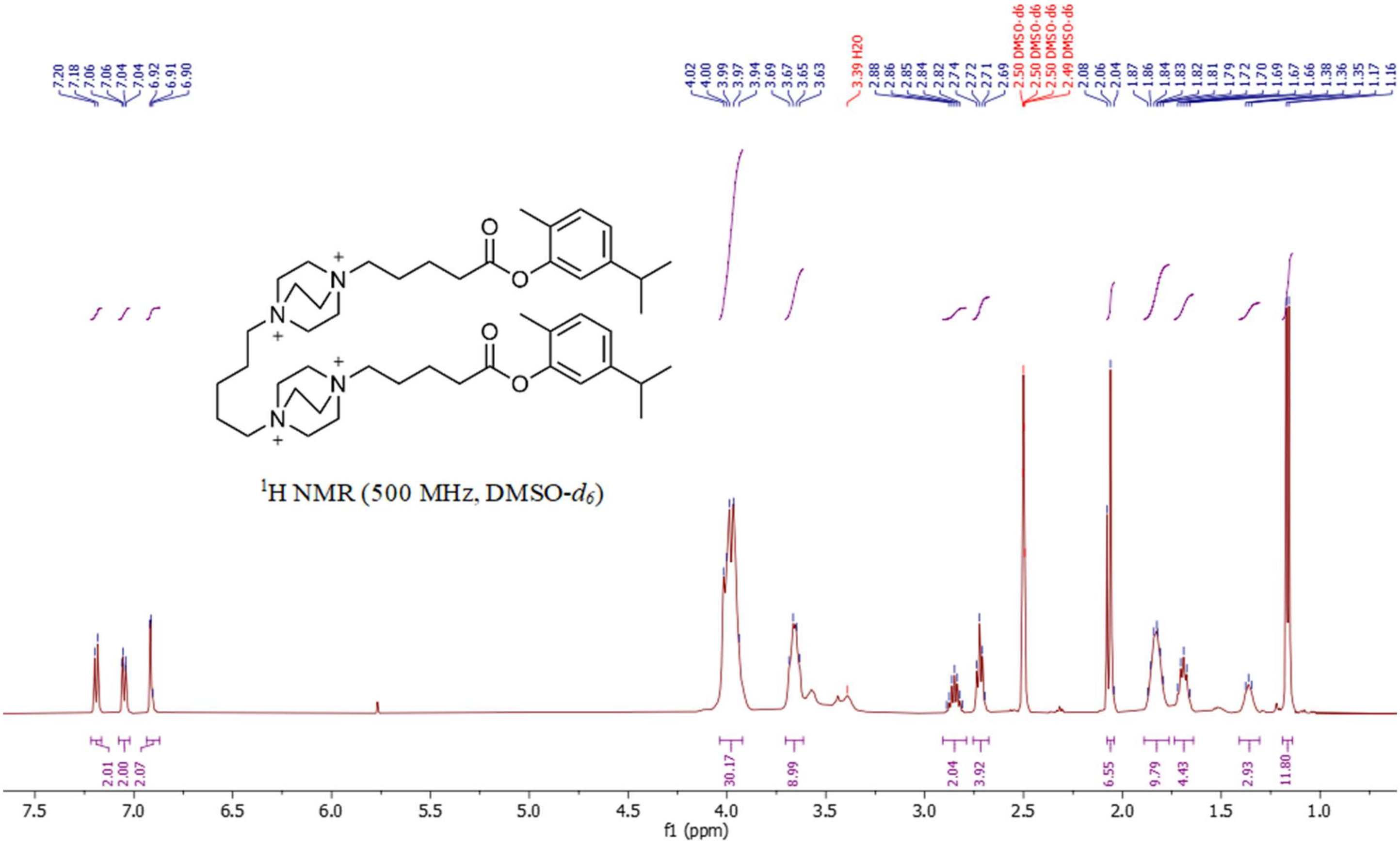

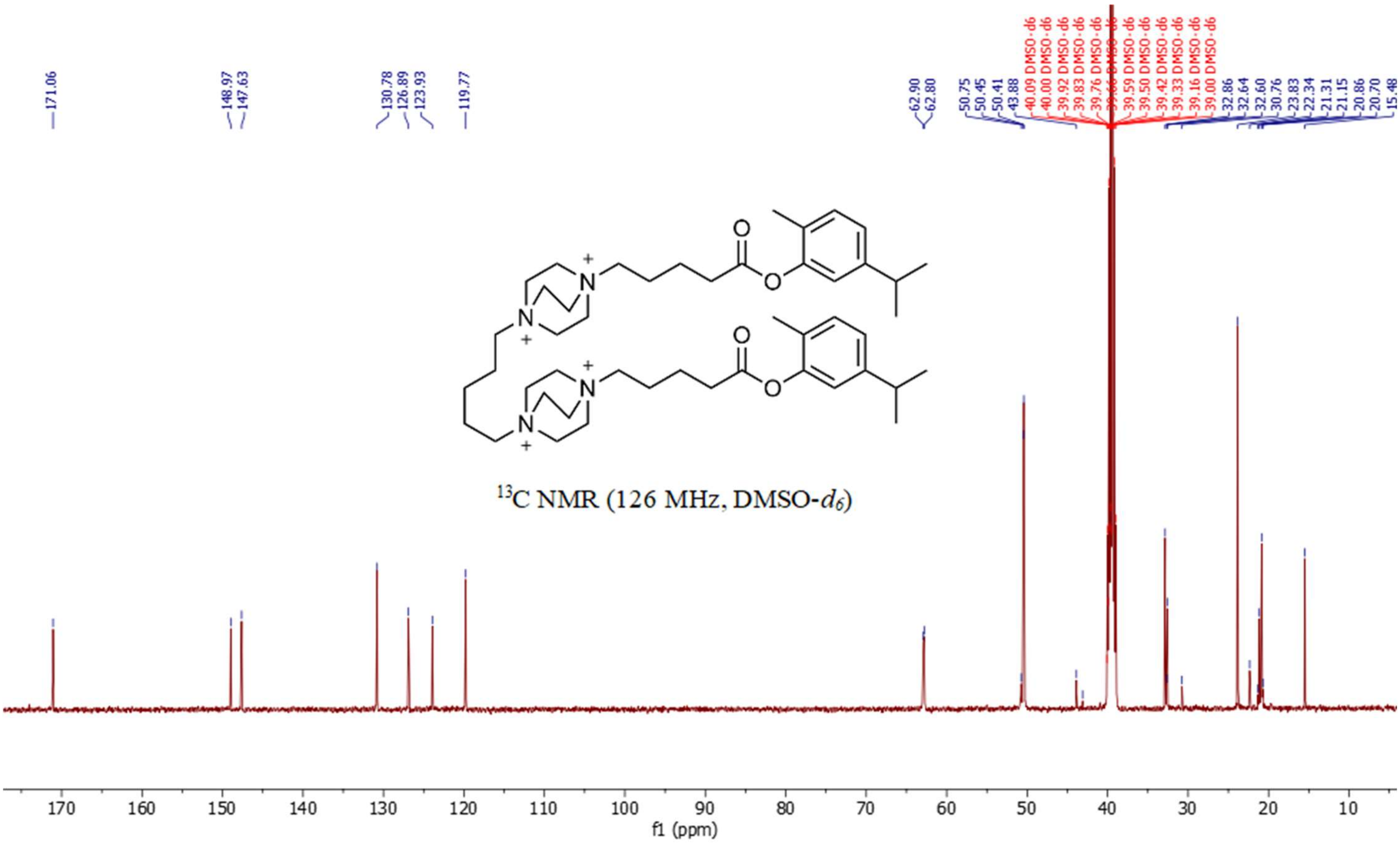

Figure S11. ^{1}H and ^{13}C NMR spectra of DL5CAR-5 (**14**).

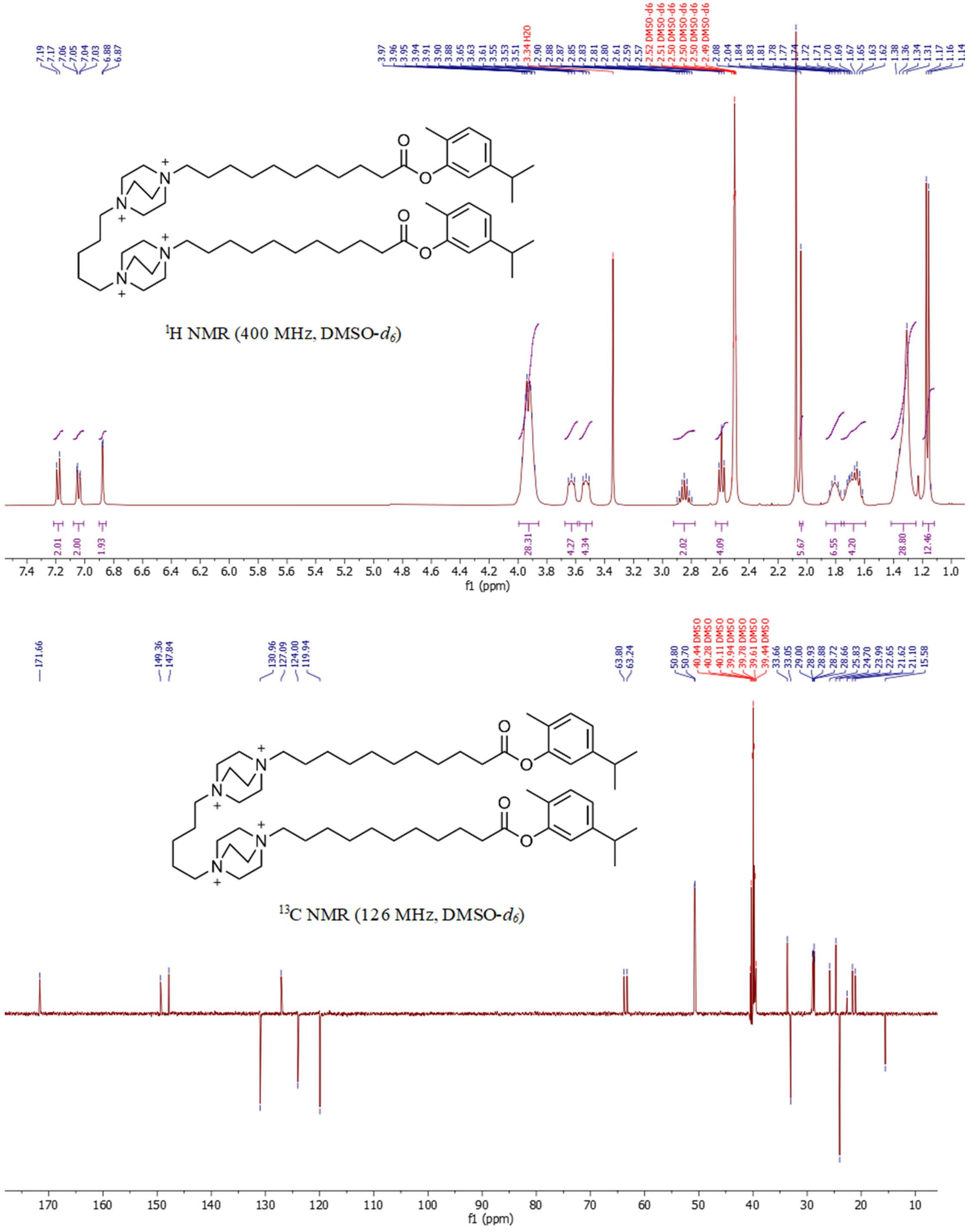

Figure S12. ^{1}H and ^{13}C NMR spectra of DL5CAR-11 (**16**).

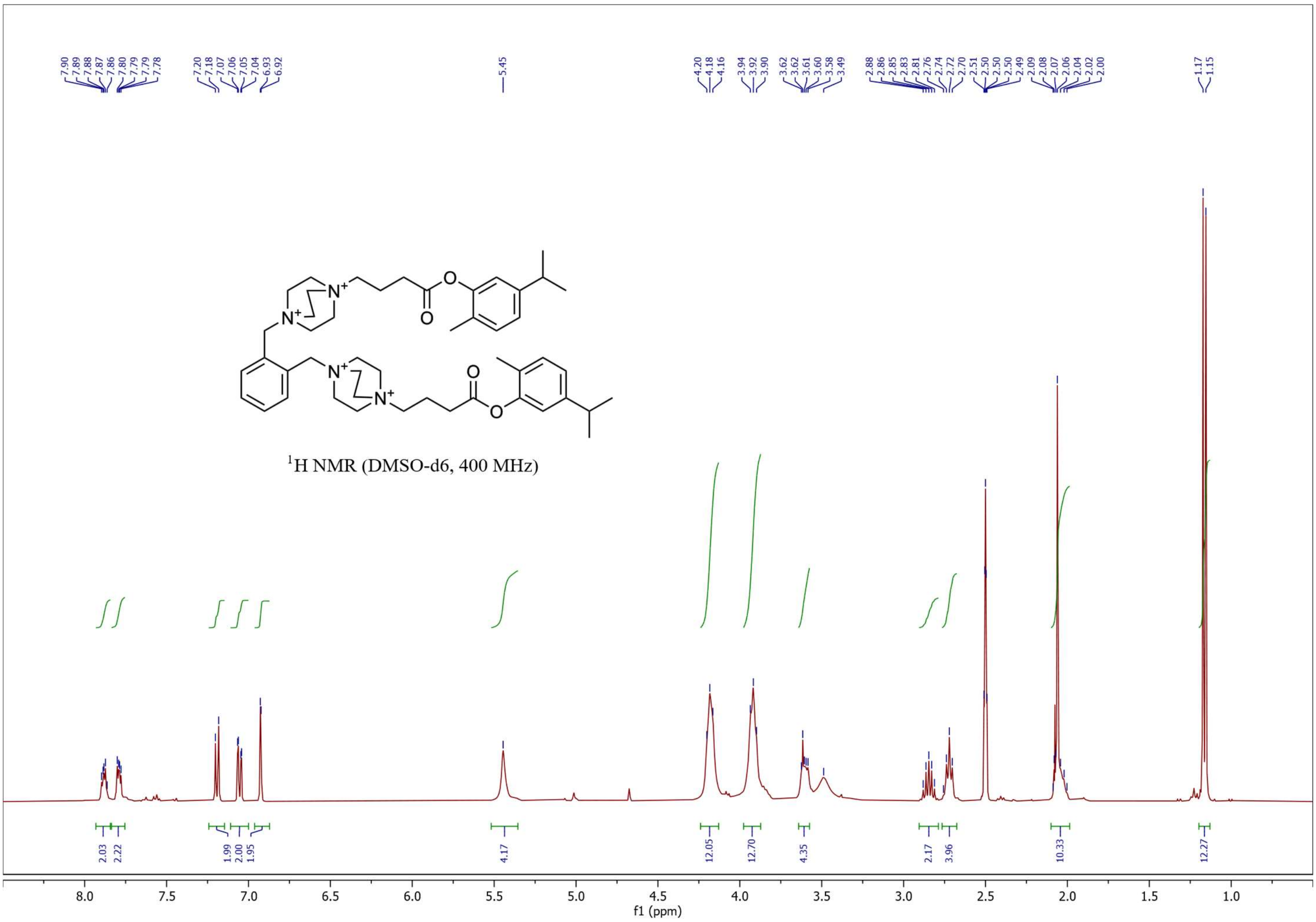

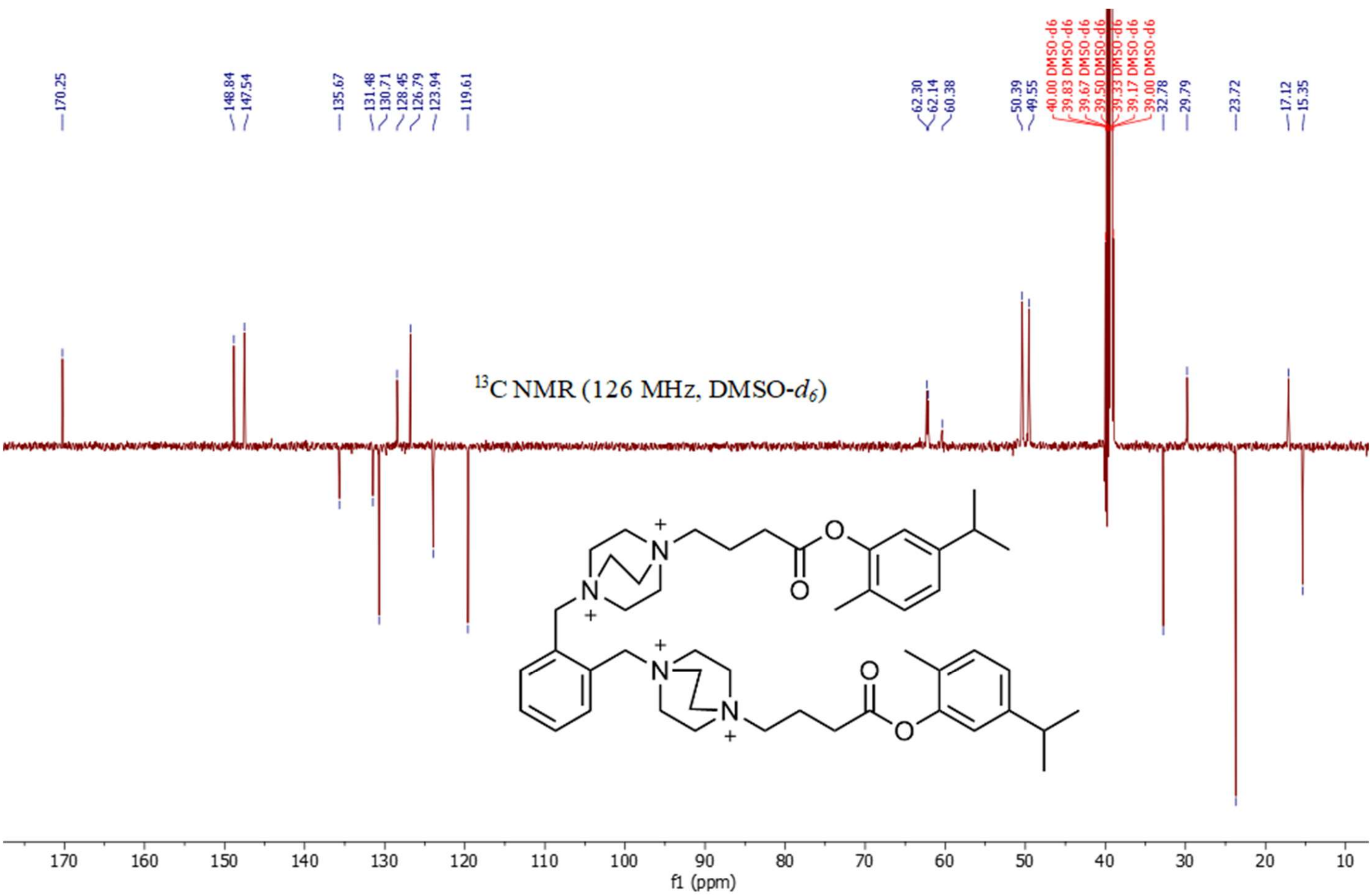

Figure S13. ^{1}H and ^{13}C NMR spectra of DLoCAR-4 (**17**).

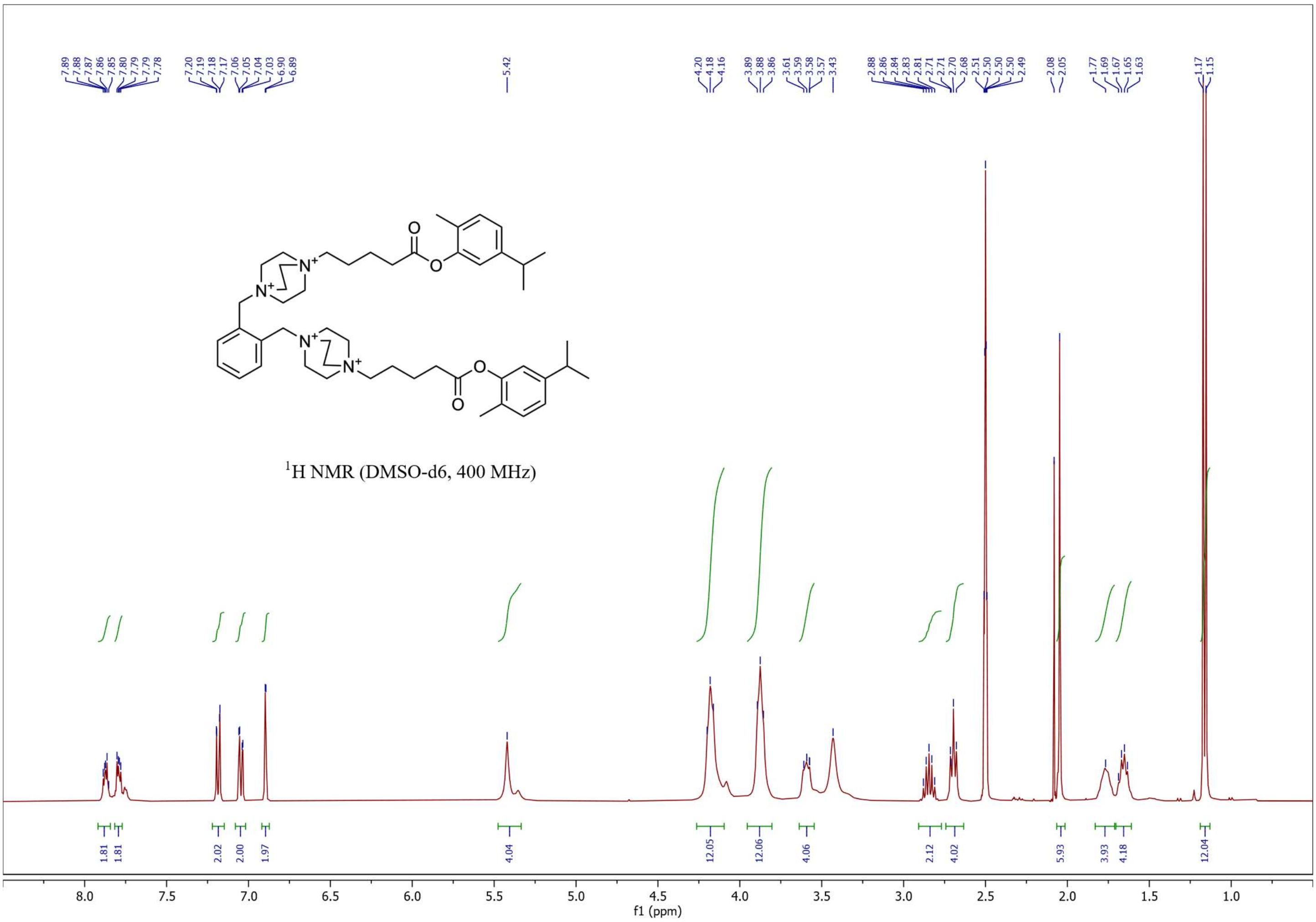

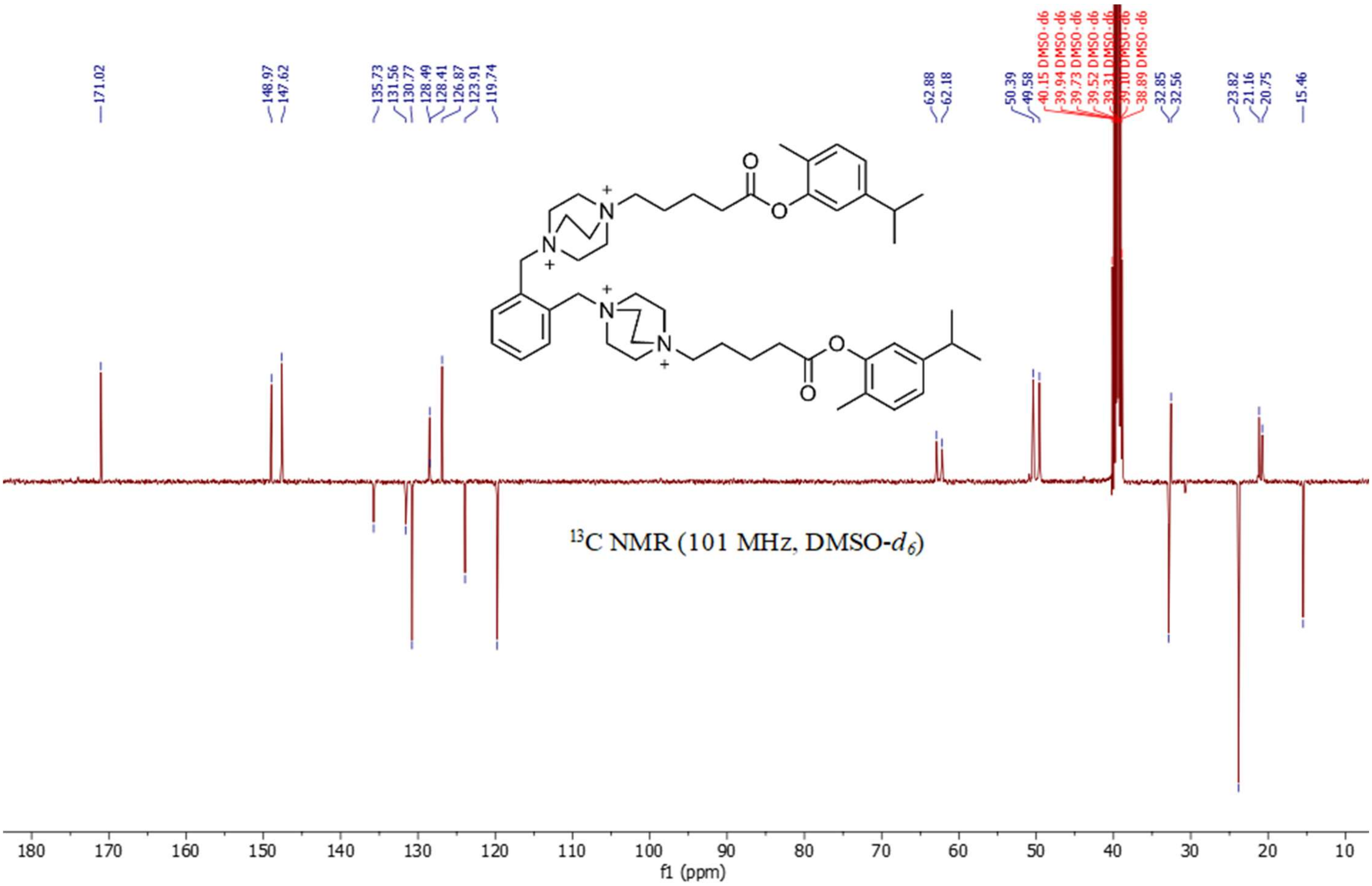

Figure S14. ^{1}H and ^{13}C NMR spectra of DLoCAR-5 (**18**).

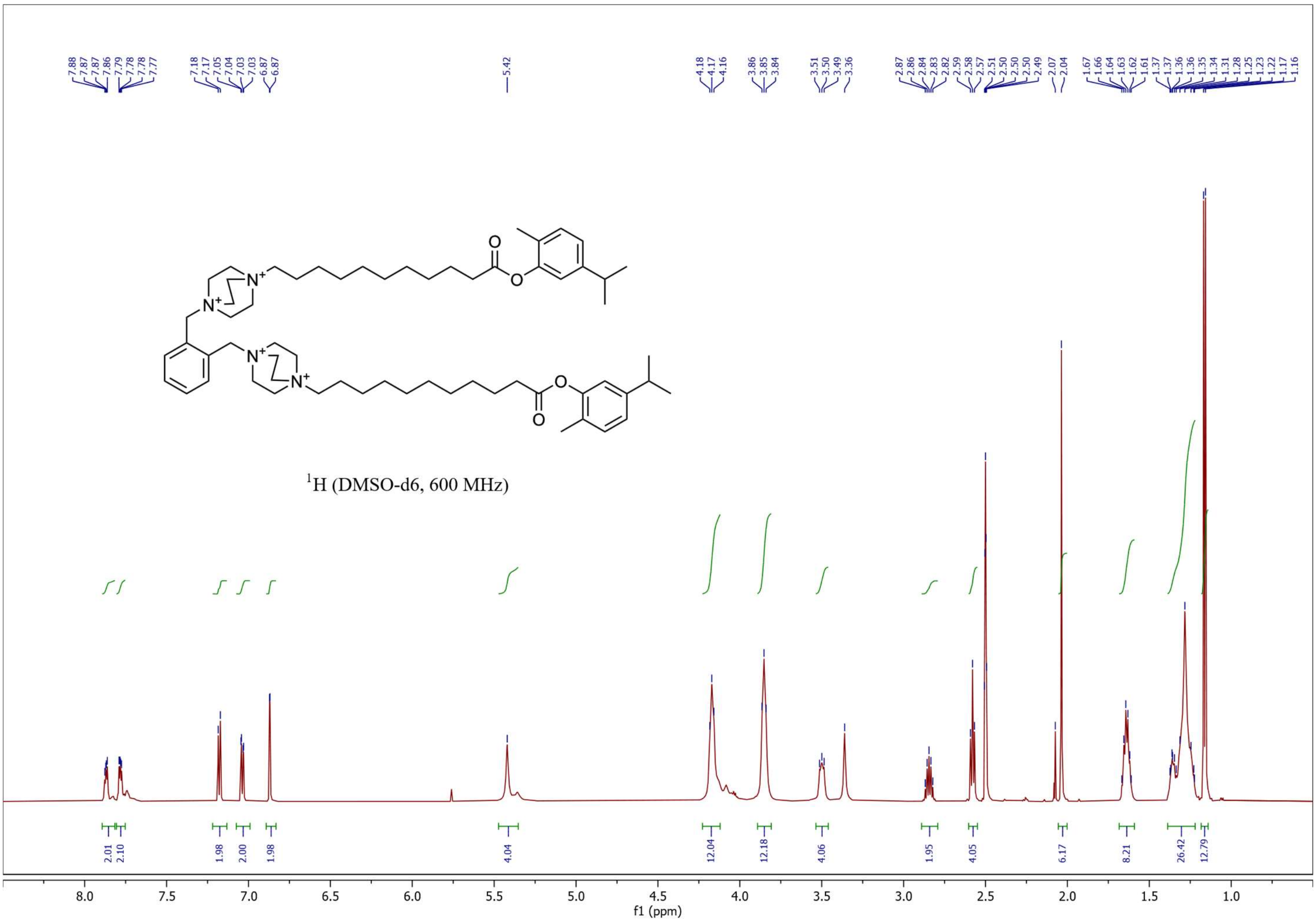

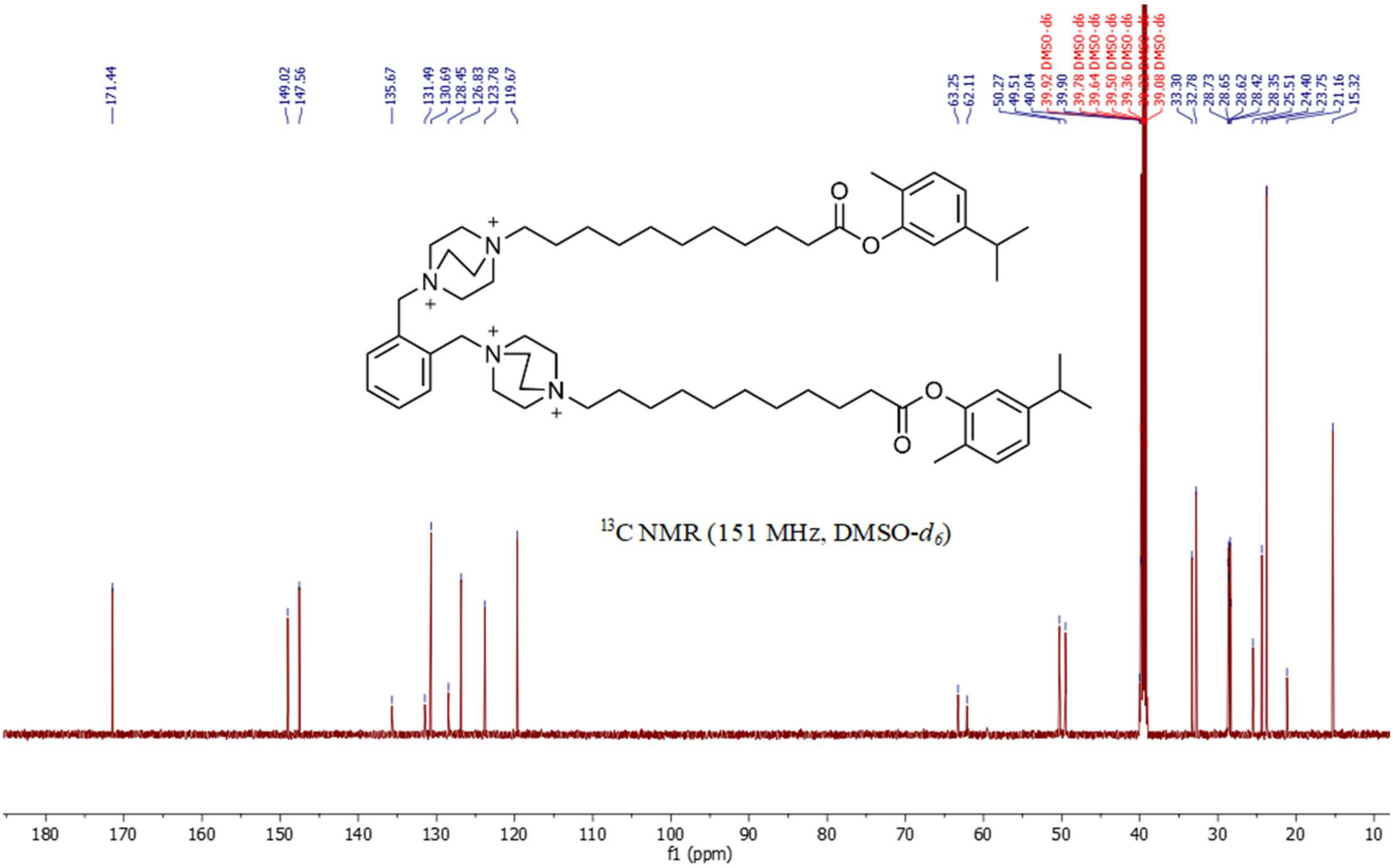

Figure S15. ^{1}H and ^{13}C NMR spectra of DLoCAR-11 (**20**).

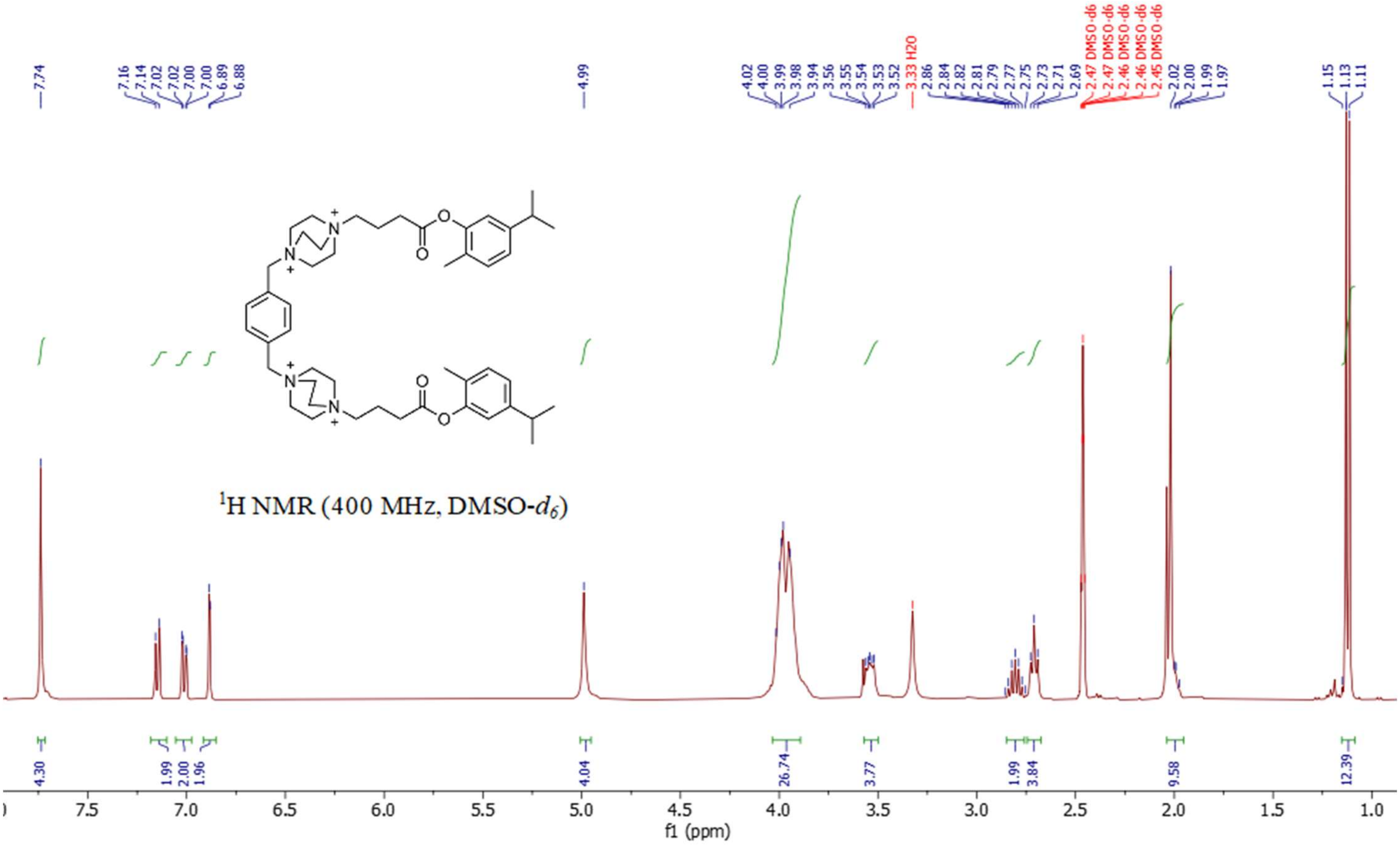

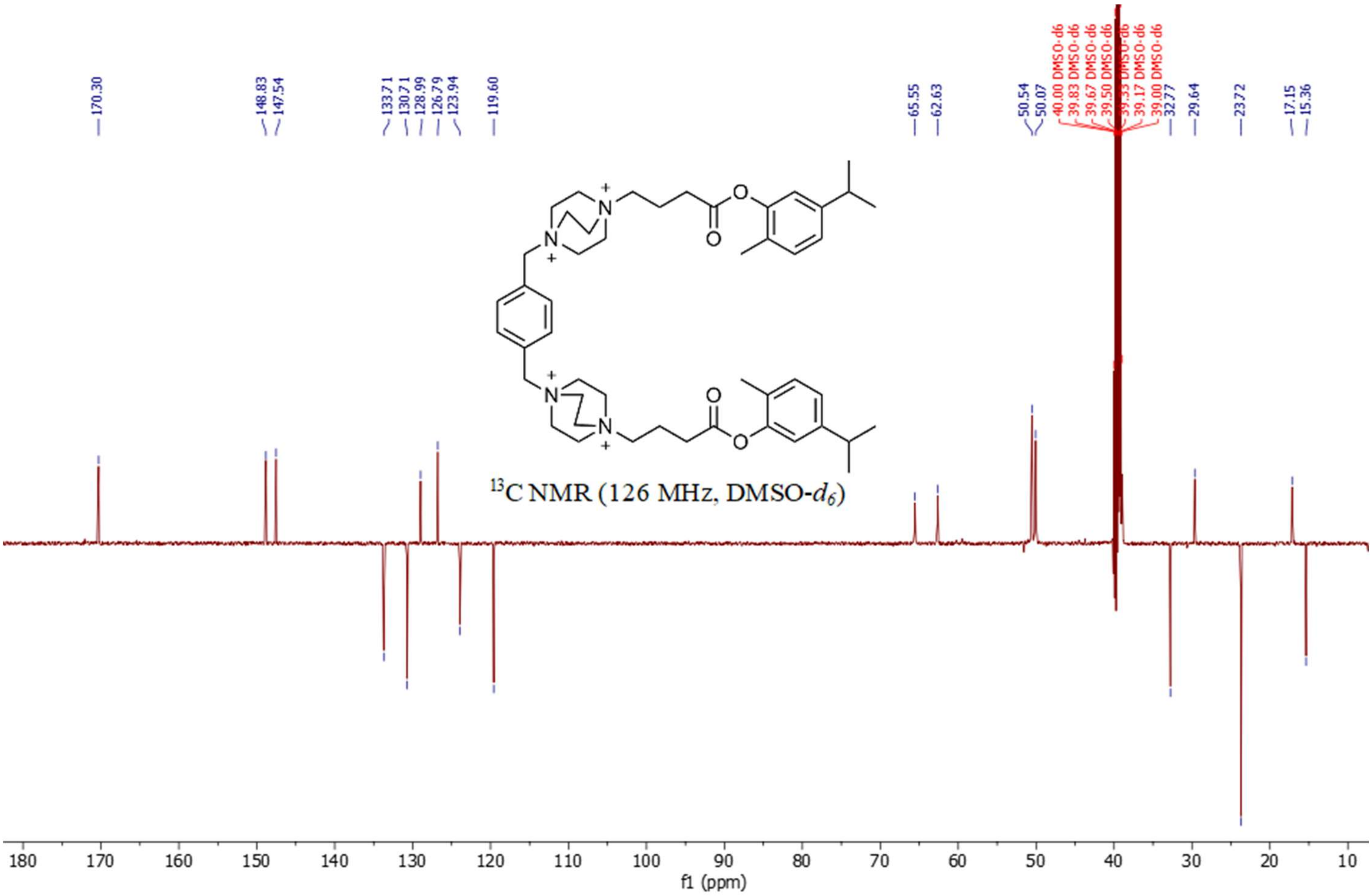

Figure S16. ^{1}H and ^{13}C NMR spectra of DLpCAR-4 (**21**).

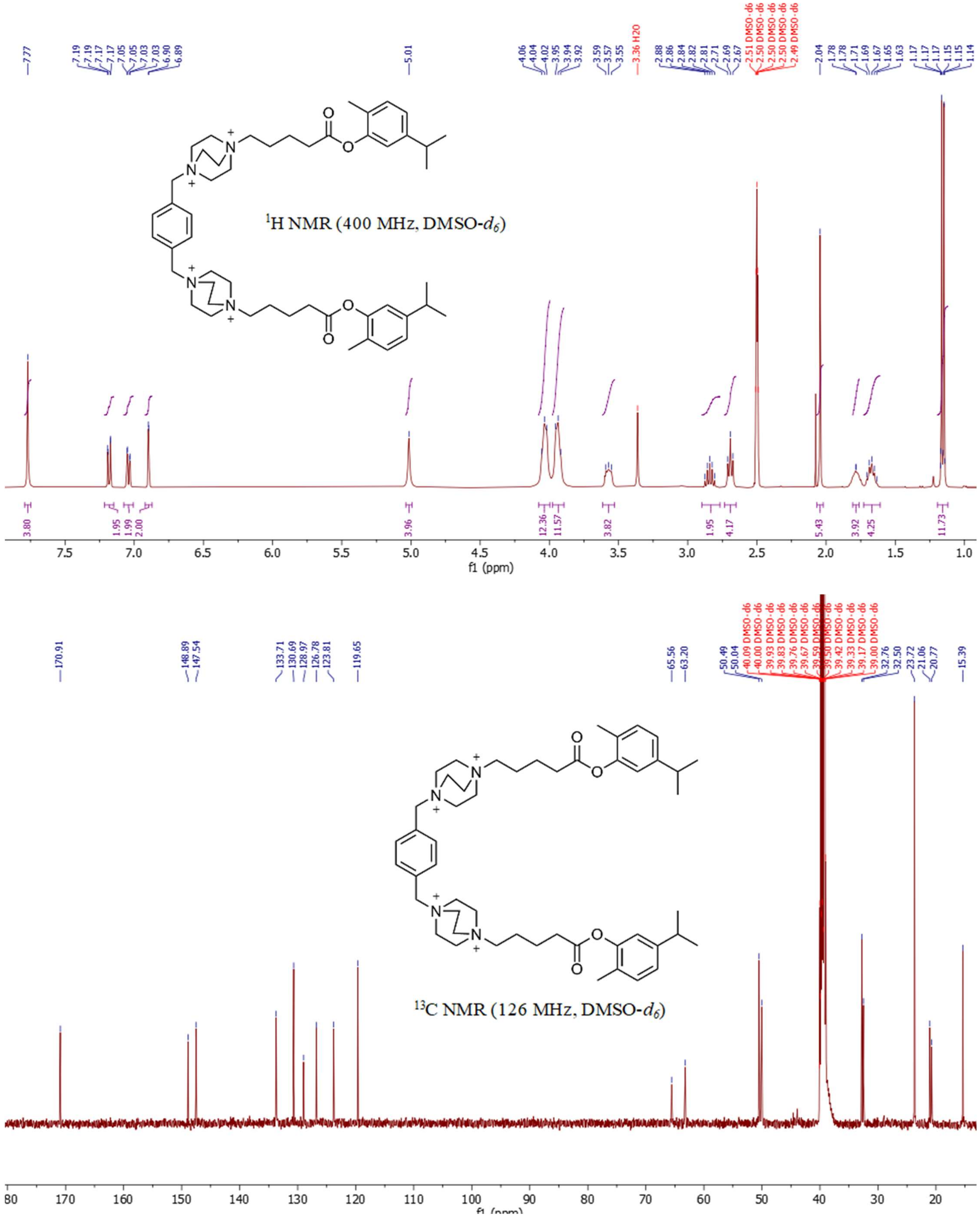

Figure S17. ^{1}H and ^{13}C NMR spectra of DLpCAR-5 (**22**).

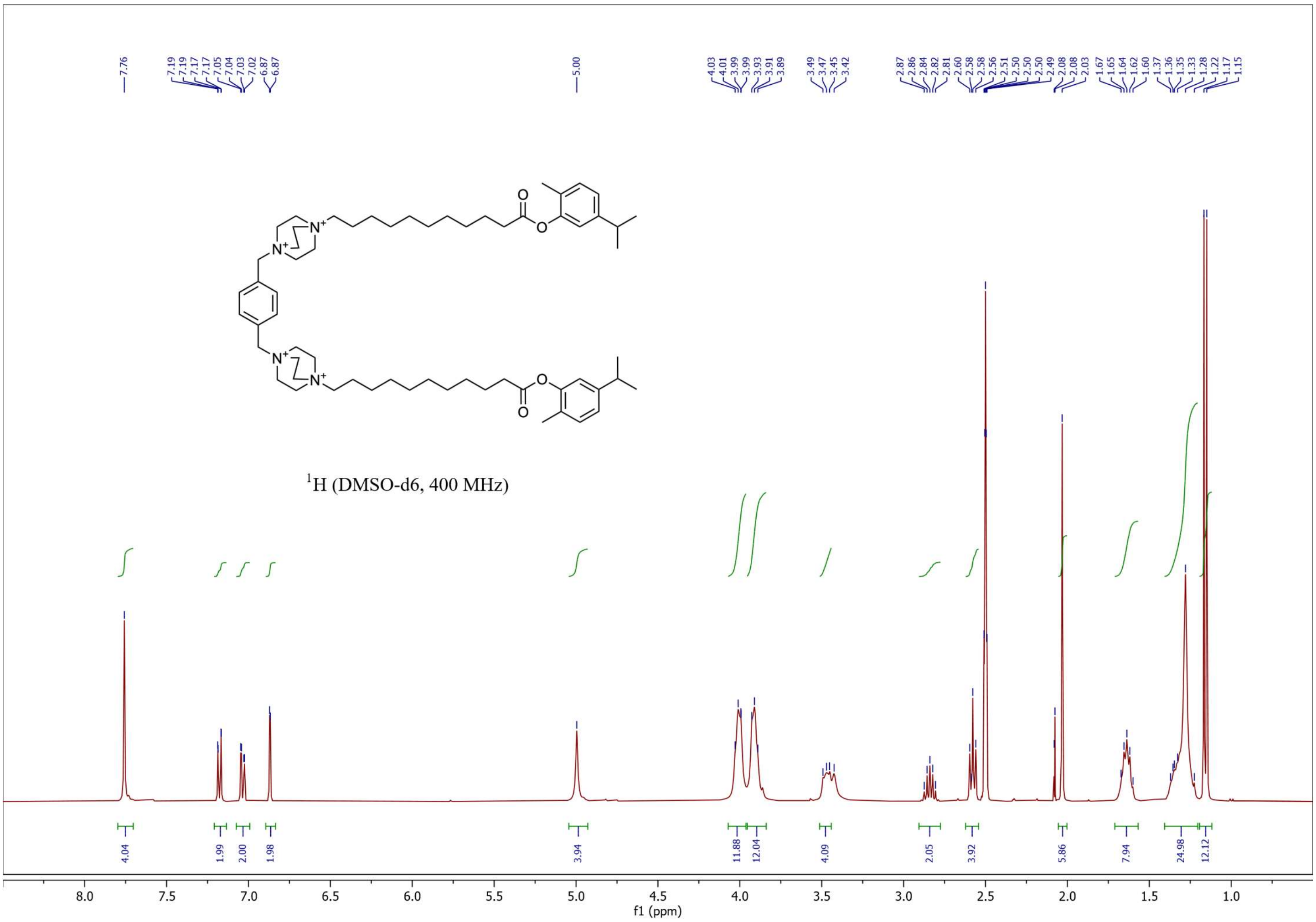

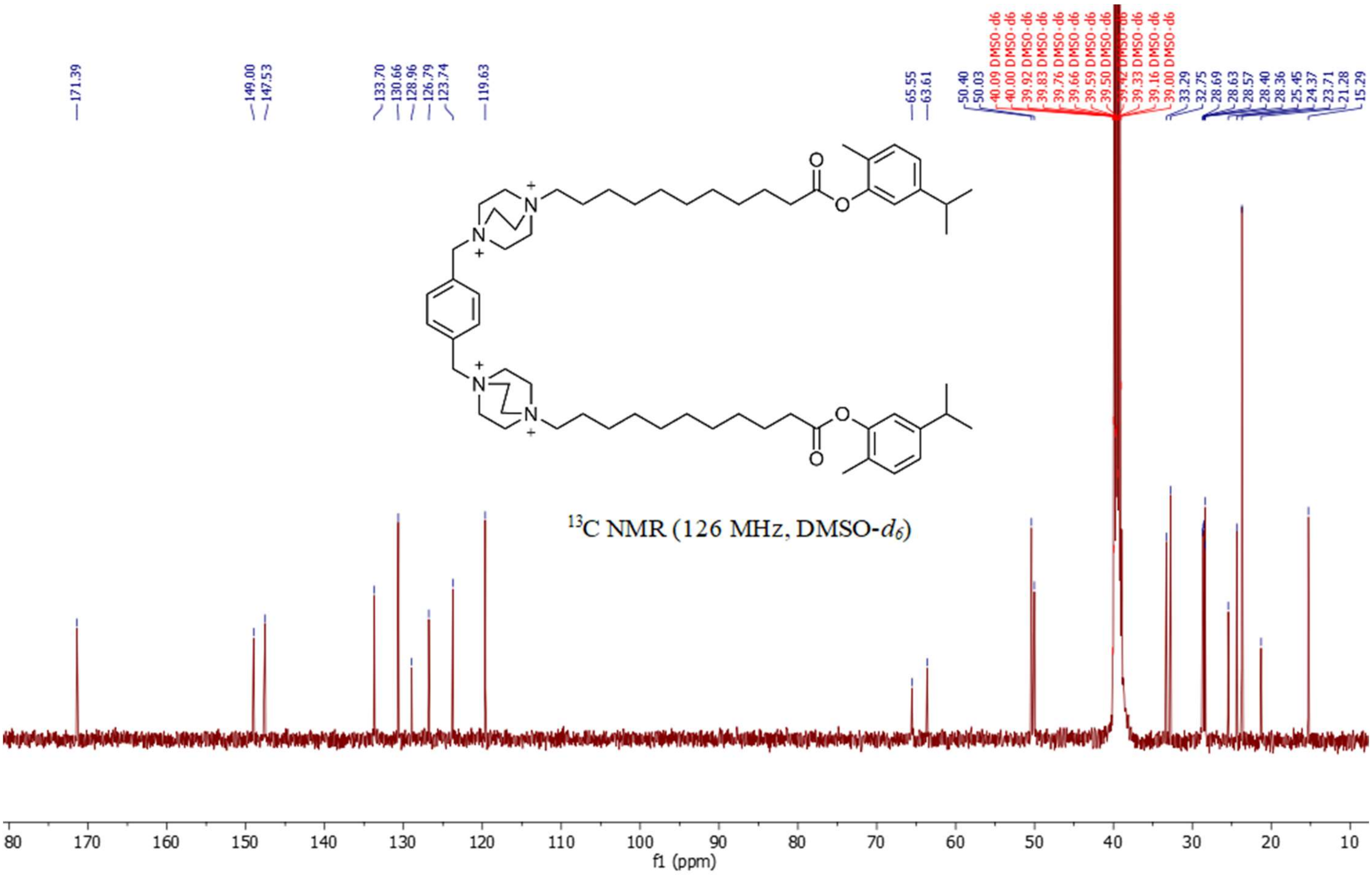

Figure S18. ^{1}H and ^{13}C NMR spectra of DLpCAR-11 (**24**).

2. MS spectra

Name	Formula (M)	Ion Formula	Found m/z	Expected m/z
(**5**) DCAR-4	$C_{20}H_{31}BrN_2O_2$	$[M - Br]^+$	331.4	331.2

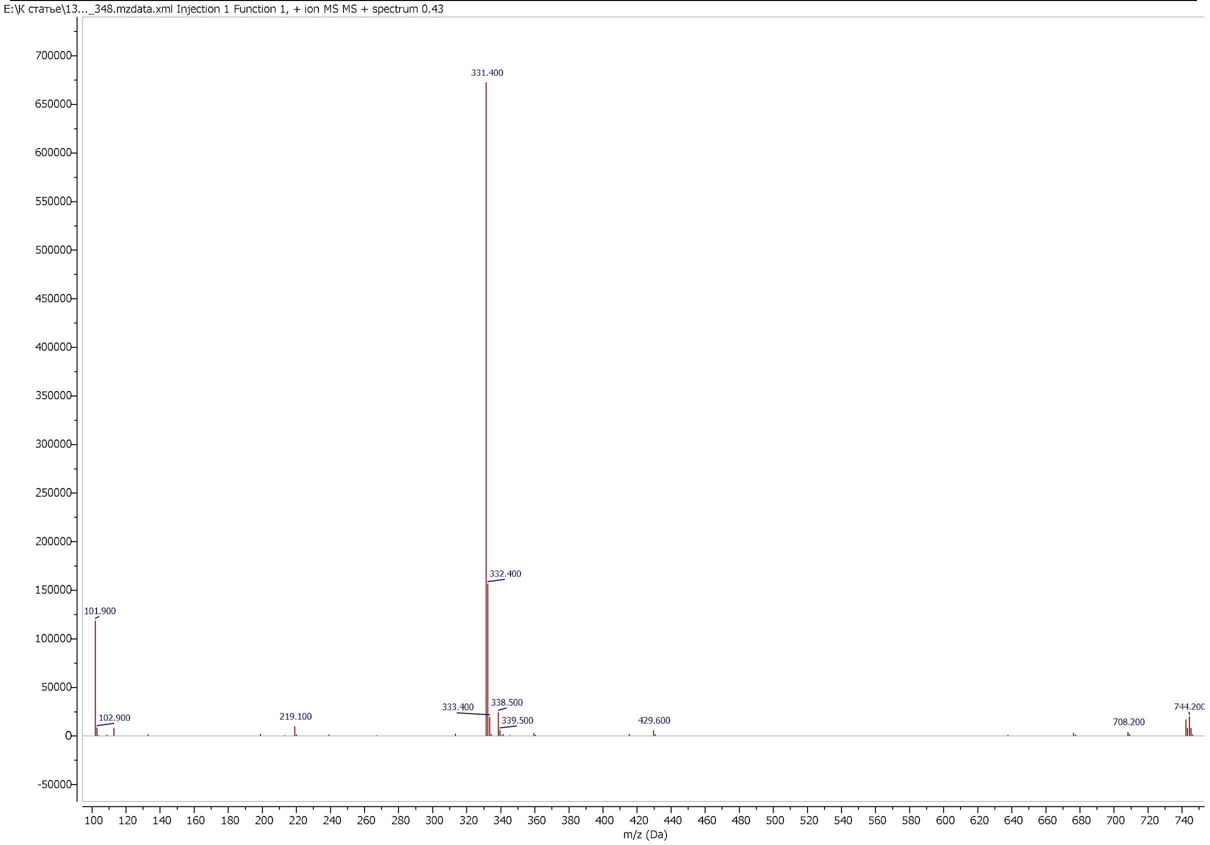

Figure S19. Mass spectrum of DCAR-4 (**5**).

Name	Formula (M)	Ion Formula	Found m/z	Expected m/z
(**6**) DCAR-5	$C_{21}H_{33}BrN_2O_2$	$[M - Br]^+$	345.4	345.3

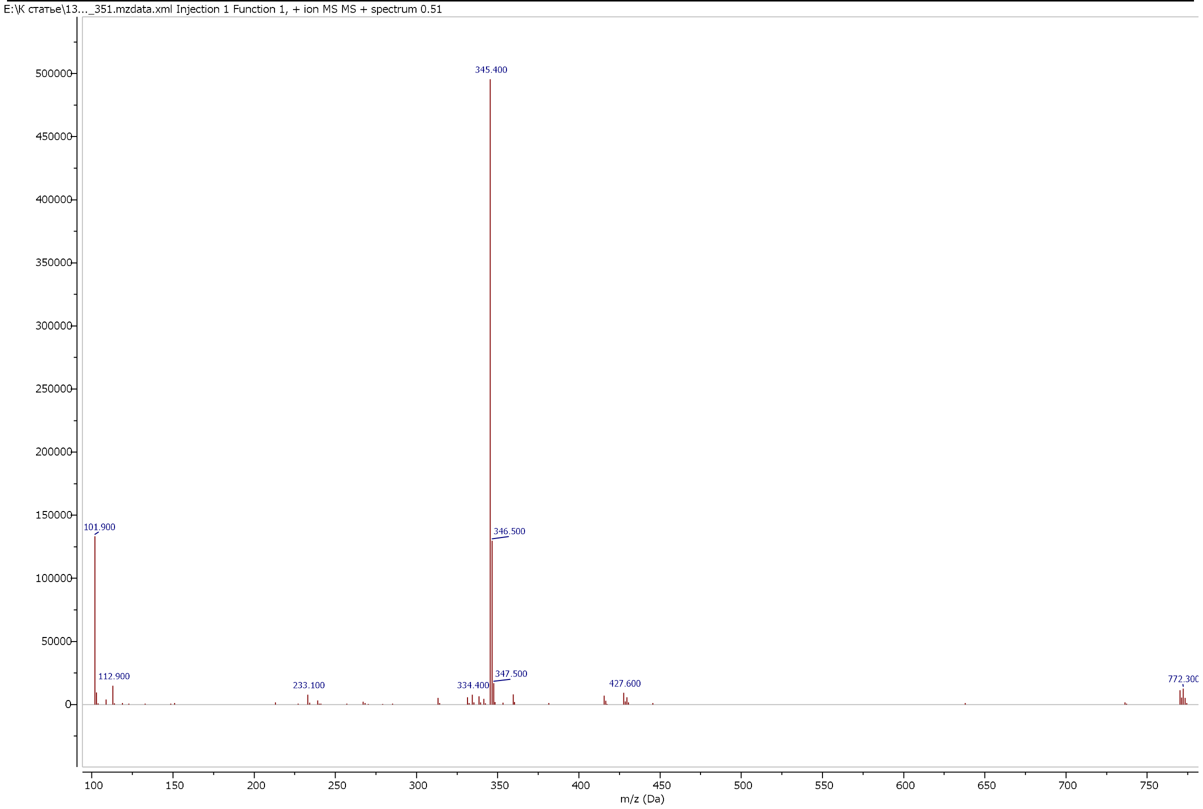

Figure S20. Mass spectrum of DCAR-5 (**6**).

Name	Formula (M)	Ion Formula	Found m/z	Expected m/z
(**8**) DCAR-11	$C_{27}H_{45}BrN_2O_2$	$[M - Br]^+$	429.7	429.4

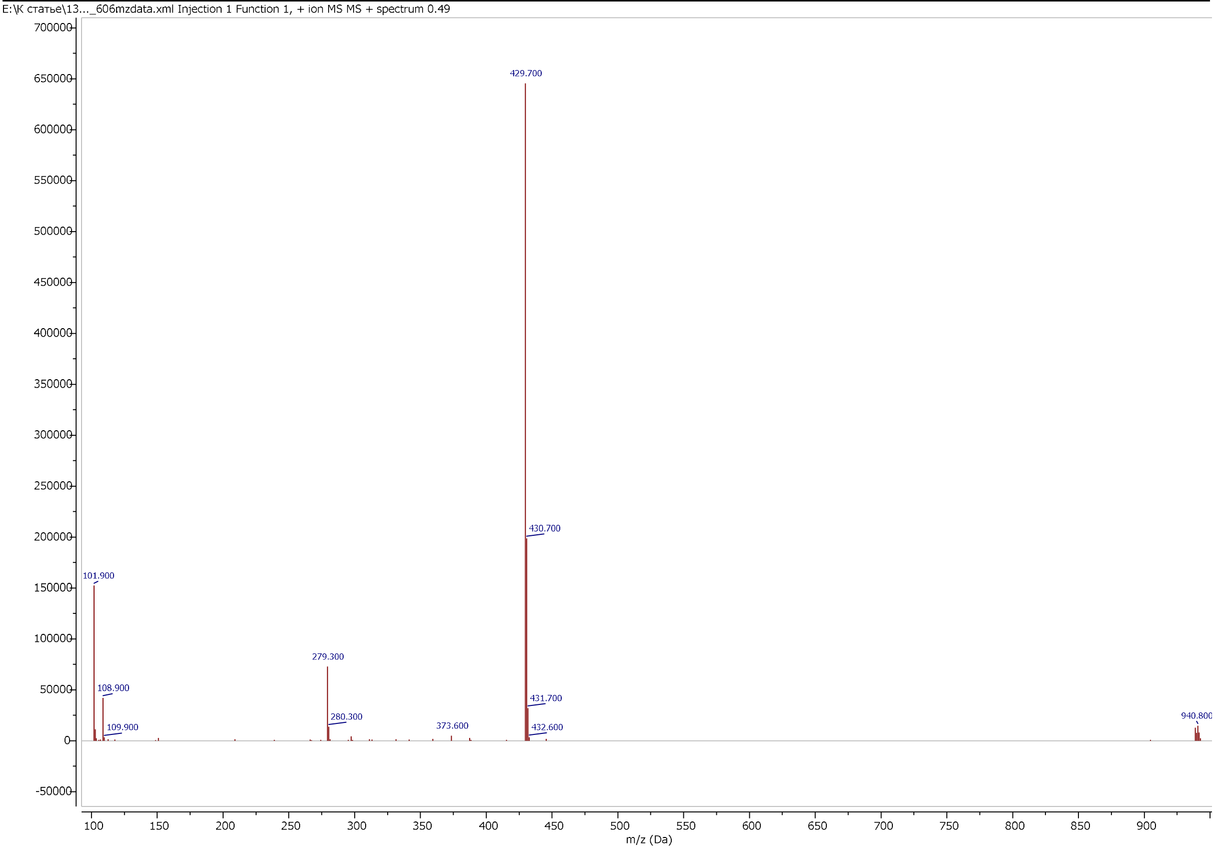

Figure S21. Mass spectrum of DCAR-11 (**8**).
